# Excitotoxicity, Oxytosis/Ferroptosis, and Neurodegeneration: Emerging Insights into Mitochondrial Mechanisms

**DOI:** 10.14336/AD.2024.0125-1

**Published:** 2025-08-01

**Authors:** Sameera Khan, Nargis Bano, Shakir Ahamad, Urmilla John, Nawab John Dar, Shahnawaz Ali Bhat

**Affiliations:** ^1^Department of Zoology, Aligarh Muslim University, Aligarh-202002, India.; ^2^Department of Chemistry, Aligarh Muslim University, Aligarh-202002, India.; ^3^School of Studies in Neuroscience, Jiwaji University, Gwalior, India; School of Studies in Zoology, Jiwaji University, Gwalior, India.; ^4^CNB, SALK Institute of Biological Sciences, La Jolla, CA 92037, USA.

**Keywords:** Mitochondrial dysfunction, Oxytosis/Ferroptosis, Excitotoxicity, Neurodegeneration, Oxidative stress, Alzheimer's disease (AD), Parkinson's disease (PD)

## Abstract

Mitochondrial dysfunction plays a pivotal role in the development of age-related diseases, particularly neurodegenerative disorders. The etiology of mitochondrial dysfunction involves a multitude of factors that remain elusive. This review centers on elucidating the role(s) of excitotoxicity, oxytosis/ferroptosis and neurodegeneration within the context of mitochondrial bioenergetics, biogenesis, mitophagy and oxidative stress and explores their intricate interplay in the pathogenesis of neurodegenerative diseases. The effective coordination of mitochondrial turnover processes, notably mitophagy and biogenesis, is assumed to be critically important for cellular resilience and longevity. However, the age-associated decrease in mitophagy impedes the elimination of dysfunctional mitochondria, consequently impairing mitochondrial biogenesis. This deleterious cascade results in the accumulation of damaged mitochondria and deterioration of cellular functions. Both excitotoxicity and oxytosis/ferroptosis have been demonstrated to contribute significantly to the pathophysiology of neurodegenerative diseases, including Alzheimer's disease (AD), Parkinson's disease (PD), Huntington’s Disease (HD), Amyotrophic Lateral Sclerosis (ALS) and Multiple Sclerosis (MS). Excitotoxicity, characterized by excessive glutamate signaling, initiates a cascade of events involving calcium dysregulation, energy depletion, and oxidative stress and is intricately linked to mitochondrial dysfunction. Furthermore, emerging concepts surrounding oxytosis/ferroptosis underscore the importance of iron-dependent lipid peroxidation and mitochondrial engagement in the pathogenesis of neurodegeneration. This review not only discusses the individual contributions of excitotoxicity and ferroptosis but also emphasizes their convergence with mitochondrial dysfunction, a key driver of neurodegenerative diseases. Understanding the intricate crosstalk between excitotoxicity, oxytosis/ferroptosis, and mitochondrial dysfunction holds potential to pave the way for mitochondrion-targeted therapeutic strategies. Such strategies, with a focus on bioenergetics, biogenesis, mitophagy, and oxidative stress, emerge as promising avenues for therapeutic intervention.

## Introduction

The understanding of mitochondrial dysfunction in the context of age-related neurodegenerative diseases has significantly advanced in recent years. Mitochondria, known for their role in energy regulation, have emerged as key players in the development of age-related disorders, including neurodegenerative diseases such as AD and PD [1]. The intricate interplay between excitotoxicity, oxytosis/ferroptosis, and mitochondrial dysfunction has been increasingly recognized as a driving force behind the pathogenesis of these diseases [2, 3]. Recent studies have highlighted the significance of mitochondrial turnover processes, particularly mitophagy and mitochondrial biogenesis, in cellular stress resistance and longevity [4-7]. However, the age-related decrease in mitophagy has been shown to impede the removal of dysfunctional mitochondria, leading to the accumulation of damaged mitochondria and the deterioration of cellular function [8, 9]. The intricate relation between mitophagy and biogenesis determines the fate of mitochondria within cells and, consequently, influences overall cellular health. Understanding these processes is essential for developing targeted therapeutic interventions that address the root causes of mitochondrial dysfunction. Excitingly, the convergence of excitotoxicity, oxytosis/ferroptosis, and mitochondrial dysfunction has opened new avenues for mitochondrion-targeted therapeutic strategies, focusing on bioenergetics, biogenesis, mitophagy, and oxidative stress. Recent studies have highlighted the significance of comprehending the intricate crosstalk among mitochondrial dysfunction, excitotoxicity, and oxytosis/ ferroptosis, as this integrated approach holds promise for enlightening effective therapeutic interventions.

Accumulating evidence suggests that mitochondrial dysfunction plays a crucial role in the pathogenesis of neurodegenerative diseases. Mitochondrial anomalies such as altered mitochondrial shape, calcium imbalance, decreased mitochondrial mass, and impaired respiration are frequently related with neurodegenerative diseases [10]. These anomalies result in decreased energy production, increased reactive oxygen species (ROS), damage to cellular components, and neuronal death [10]. Furthermore, mitochondrial dysfunction results in apoptosis, autophagic dysregulation, and DNA damage, all contributing to the progression of neurodegenerative disorders [11]. Remarkably, 17% of the clinical therapies focus on signaling pathways and processes connected to mitochondrial dysfunction as a possible and prospective therapeutic intervention for treating neurodegenerative diseases [12, 13]. The amalgamation of these insights not only has advanced our comprehension of neurodegenerative diseases but also drives us toward the prospect of transformative therapeutic strategies, aiming to mitigate the intricate interplay of mitochondrial dysfunction, excitotoxicity, and oxytosis/ferroptosis in the context of age-related neurodegenerative diseases.

Mitochondria play a crucial role in regulating cell death pathways, including oxytosis/ferroptosis and glutamatergic excitotoxicity [14]. Dysfunction in these processes can lead to the accumulation of ROS and lipid peroxides, ultimately resulting in cell death [14]. Oxytosis/Ferroptosis can be triggered by various factors, such as depletion of glutathione or inhibition of the cystine/glutamate antiporter system [15]. Oxytosis/ Ferroptosis is involved in oxidative-stress induced cell death via the generation of ROS through iron dependent enzymatic reactions, ultimately leading to lipid peroxidation and membrane damage in several neurodegenerative diseases [16].

Glutamatergic excitotoxicity, on the other hand, involves the excessive activation of glutamate receptors, leading to an influx of calcium ions and subsequent cell death [17]. Glutamatergic excitotoxicity also results in the production of ROS, further exacerbating oxidative stress within the cell [18]. This exacerbated ROS contributes to lipid peroxidation and membrane damage, ultimately perpetuating the cycle of cell death [19]. The combination of iron-dependent lipid peroxidation and glutamatergic excitotoxicity can lead to significant damage to cells and tissues, causing a cascade of detrimental effects that can ultimately result in cell death [20]. Both of these pathways interact with each other and with mitochondrial function, highlighting the complex and intertwined nature of cell death mechanisms in neurodegenerative disorders [20]. Understanding the role of mitochondria in these processes may provide potential targets for therapeutic interventions aimed at protecting neurons from cell death and slowing the progression of neurodegenerative diseases such as AD, PD, and HD.

Therefore, a comprehensive review of the latest studies in this field is essential for understanding the full scope of the involvement of mitochondrial dysfunction in age-related neurodegenerative diseases and identifying promising therapeutic targets. Emerging scientific innovations using mitochondrion-targeted therapeutic approaches offer new hope for the development of effective treatments for these debilitating conditions.

## Mitochondrial Dysregulation in Neurodegenerative Pathophysiology

**Figure 1.**
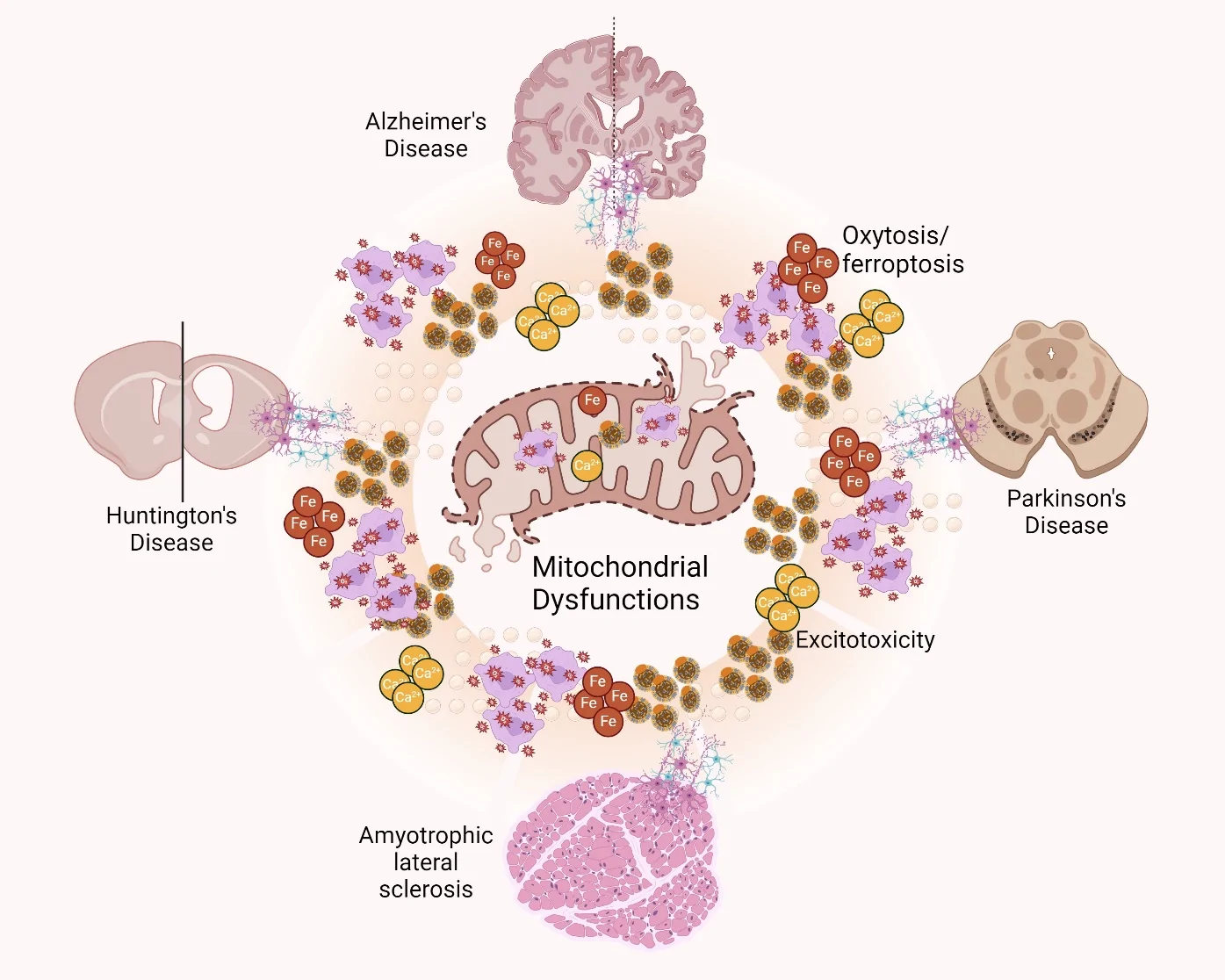
**Mitochondrial Dysfunction in Neurodegenerative Pathogenesis**. Schematic representation illustrating the central role of mitochondrial dysfunction in the pathogenesis of neurodegenerative disorders. Mitochondrial dysfunction, accompanied by increased calcium, lipid peroxidation, elevated reactive oxygen species (ROS), and iron accumulation. These factors collectively serve as a link for key mechanisms contributing to neurodegeneration. The figure highlights the interconnected nature of these pathways and their collective influence on the pathophysiology of neurodegenerative diseases.

Mitochondria play crucial roles in energy production, cellular metabolism, intracellular signaling, apoptosis, and free radical production [21, 22]. Studies have shown that mitochondrial dysfunction in neurodegenerative disorders is associated with a range of abnormalities, including altered mitochondrial morphology, calcium imbalance, reduced mitochondrial mass, and impaired mitochondrial respiration [21, 23-29]. These abnormalities can lead to decreased energy production and increased production of ROS, which can damage cellular components [21, 23-29]. Additionally, mitochondrial dysfunction can result in enhanced apoptosis, autophagy, and mitochondrial DNA damage [12, 14-20, 21]. These cellular dysfunctions contribute to the development and progression of neurodegenerative disorders by causing neuronal dysfunction and cell death. For example, in AD, mitochondrial dysfunction has been observed early in the disease process, with reductions in mitochondrial respiration and an increase in β-amyloid [30]. In PD, genes such as Parkin and PINK1, associated with mitochondrial quality control, have been found to be linked to the disease [31]. These examples highlight the important role of mitochondrial dysfunction in the pathogenesis of neurodegenerative disorders. Further, mutant huntingtin (mHTT) directly interacts with the outer mitochondrial membrane (OMM), and causes mitochondrial dysfunction (Fig. 1) [27, 28]. In addition, mHTT reduces the expression of peroxisome proliferator-activated receptor gamma coactivator 1-alpha (PGC-1α), a key protein involved in mitochondrial homeostasis [32], which in turn prevents the activation of downstream signaling molecules, like nuclear respiratory factors, NRF-1 and NRF-2, estrogen related receptor α (ERRα), and the nuclear receptors, PPARα, PPARδ, and PPARγ [33]. These transcription factors govern the expression of several nuclear-encoded mitochondrial genes, such as complexes I-V, Cyt c, and the mitochondrial transcription factor A (TFAM) [33]. Additionally, PGC-1α-NRF1/NRF2-TFAM pathway is significantly associated with mitochondrial biogenesis, whereas ectopic PGC-1α expression results in neuroprotection in transgenic HD mice and the 3-NPA mouse model of HD [34]. Moreover, synaptic mitochondria are more susceptible to damage and stress than mitochondria in the cell body [35]. The injured synaptic mitochondria are unable to provide sufficient energy for synaptic vesicle transmission, resulting in cognitive and learning failure [35]. Further, alprazolam-induced mitochondrial dysfunction significantly increased the number of differentially expressed proteins, including mitochondrial proteins involved in mitochondrial dynamics (FIS1 and OPA1), the mitochondrial respiratory chain (complexes I (Ndufc2, Ndufs5, and ND4), complex IV (COX 4, COX 6, and ATP 8), apoptosis (BCL2), and mitochondrial autophagy (ATG3 and LC3) in AD, suggesting a link between mitochondrial dysfunction and cognitive impairment [29]. However, targeting mitochondrial dysfunction with NSI-189, enhances total neurite growth and mitochondrial functions, further strengthening the involvement of mitochondria in cognition [29]. CDK5 phosphorylates cell cycle exit and neuronal differentiation 1 (CEND1), a presynaptic mitochondrial protein that promotes neuronal differentiation by decreasing the levels of cell cycle protein S10, consequently leading to its degradation [35]. Decreased levels of CEND1 induce dynamin-related protein (Drp1) overexpression and excessive mitochondrial fission, which compromises mitochondrial function and energy production, ultimately leading to cognitive impairments in a 5xFAD mouse model of AD (Fig. 1) [35]. In addition to Drp1, CEND1 also interacts with other mitochondrial proteins, such as OPA1, ATP5O, and VDAC3, all of which play crucial roles in mitochondrial dynamics and oxidative phosphorylation [36-38].

Several studies have shown the involvement of voltage-dependent anion channel-1 (VDAC1) in mitochondrial dysfunction in neurodegenerative disorders [39-46]. VDAC1 is involved in mitochondria-mediated apoptosis [40-46]. Stress and apoptosis inducers cause VDAC1 overexpression and oligomerization, which results in the formation of large channels that release proapoptotic proteins to the cytosol [40-46]. Further, VDAC1 switches from supporting critical metabolic processes to promoting apoptosis by binding to apoptotic regulatory proteins like Bcl-xL, Bcl-2, and hexokinase [40-46]. Aβ-induced VDAC1 over-expression in the neuropils surrounding Aβ plaques is associated with mitochondrial dysfunction, leading to apoptosis and neuroinflammation [39]. However, treatment with VBIT-4, a small molecule inhibiting the oligomerization of VDAC1, mitigates mitochondrial dysfunction and apoptosis [39]. Similarly, the interaction of α-synuclein with VDAC1 increases its permeability for mitochondrial calcium uptake, which is associated with mitochondrial dysfunction and cell death (Fig. 1) [47]. Further, after binding to hexokinase, VDAC1 promotes downstream events on the mitochondrial surface that induce NLRP3 inflammasome assembly and activation, resulting in inflammation and cell death [39, 48]. By restoring mitochondrial oxidative phosphorylation and inhibiting dependency on anaerobic respiration, VBIT-4 promotes a neuroprotective phenotype in microglia and astrocytes, consequently attenuating neuroinflammation [39, 49, 50]. Additionally, lipopolysaccharide (LPS) induced-mitochondrial dysfunction exacerbated inflammation, with subsequent accumulation of α- synuclein oligomers in enriched mesencephalic neuronal cultures [50]. Moreover, LPS failed to induce inflammation in human pluripotent embryonal carcinoma cells with deficient mtDNA (NT2-Rho0) without functional mitochondria [50].

Mitochondrial dysfunction disrupts the integrity of the mitochondrial membrane, causing mitochondrial ligands to be released into the cytoplasm or outside of the cell. LPS activates innate immune responses by inducing mitochondrial impairment, evidenced by the increased mitochondrial fragmentation concomitant with the release of cardiolipin, mtDNA, ROS and ATP in pure mesencephalic neurons [50, 51]. These molecules act as DAMPs and therefore activate inflammatory processes in neurodegenerative diseases [50, 51]. Moreover, the loss of the immune-privileged state of extracellularly damaged mitochondria is associated with enhanced release of damaged mitochondria from microglia, and damaged extracellular mitochondria promote disease propagation by serving as an innate immune response effector, targeting adjacent neurons and astrocytes [52]. Enhanced Drp1/Fis1-induced mitochondrial fission in activated microglial causes the production of damaged and fragmented mitochondria, which are discharged from these cells, thus eliciting an innate immune response [52, 53]. The Drp1/Fis1 inhibitory peptide P110 decreases mitochondrial fission and the subsequent release of damaged mitochondria from microglia, reducing astrocyte activation and safeguarding neurons from innate immune attack [52, 54-56].

Additionally, studies have supported the association of functionally impaired mitochondria with ROS generation, oxidative stress and neurodegeneration [57, 58]. Mitochondrial malfunction, including increased mtROS generation, is linked to neuronal apoptosis (Fig. 1) [57]. Further, a study found that knocking down TFAM in the nigrostriatal pathway in a MitoPark mouse model led to mitochondrial dysfunction, evidenced by the reduced ATP levels, *m*-aconitase activity, elevated oxidative stress, and modified mitochondrial morphology suggesting that TFAM plays a critical role in maintaining mitochondrial function [58]. Moreover, treatment with mito-apo, a mitochondrion-targeted apocynin (conjugated to a triphenylphosphonium cation moiety through an alkyl chain with differing chain lengths [C_2_-C_11_ carbon atoms]), decreased striatal neurotransmitter depletion, nigrostriatal degeneration, and progressive motor deficits by restoring functional mitochondria by mitigating oxidative stress and rescued mitochondrial bioenergetics, further suggesting the involvement of impaired mitochondria in ROS generation and oxidative stress [58, 59]. In Aβ_1-42_-induced in vivo and in vitro AD models, activation of the JNK-RIP1-STAT3 pathway results in mitochondrial dysfunction and subsequent mtROS production. Pretreatment with Ginkgo biloba extract 761 (EGb761) reportedly alleviated mitochondrial impairment and ROS production by inhibiting downstream apoptotic signaling in an AD model [60]. Similarly, insulin and IGF-1 improved mitochondrial functions and reduced mHtt-induced mtROS production by activating the PI-3K/Akt signaling pathway, independent of nuclear factor erythroid 2 related factor 2 (Nrf2) transcriptional activity but in a mitochondrial-encoded complex IV subunit and Akt dependent manner [61]. Further, in HD mice, abnormal Drp1-mediated mitochondrial fragmentation in the striatum causes mitochondria to move away from the endoplasmic reticulum (ER), disrupting the ER-mitochondrial association, resulting in decrease efflux of calcium from ER into the mitochondria and increased mitochondrial superoxide generation, mitochondrial dysfunction and degeneration of the striatal neurons [62]. Accordingly, Mdivi-1 treatment inhibited Drp1 activity and restored ER-mitochondrial connections, mitigated mitochondrial dysfunction, and promoted calcium homeostasis (Fig. 1) [62]. Furthermore, while physiological ROS levels are required for cellular signaling, however, heightened and prolonged ROS exposure not only damages the cellular macromolecules such as DNA, lipids, and proteins, but also accelerates the mutation rate of mitochondrial DNA (mtDNA) [63, 64]. Further increased expression and activation of NADPH oxidase 4 by hampering mitochondrial respiration and ATP production in astrocytes, results in mtROS generation and neurotoxicity in AD [57]. Overall, in the last few decades, cumulative data suggests that mitochondrial malfunction and oxidative stress lead to the physiological decline that accumulates with age and in age-related neurodegeneration.

There is overwhelming evidence of impaired mitochondrial function in other neurodegenerative diseases such as HD, amyotrophic lateral sclerosis (ALS) and multiple sclerosis (MS) [32]. Recent studies have highlighted the importance of mitochondrial dynamics, including processes such as fusion, fission, distribution, and trafficking, in the pathogenesis of these diseases [65]. Mitochondrial dysfunction is a prominent feature in HD, characterized by mitochondrial loss, altered dynamics including increased fission and reduced fusion, and changes in mitochondrial size and number in striatal neurons correlating with disease severity [65]. Both symptomatic HD patients and asymptomatic mutation carriers exhibit impaired mitochondrial function, evidenced by slowed phosphocreatine recovery and oxidative function [66]. In amyotrophic lateral sclerosis (ALS), mitochondrial dysfunction is a central aspect of its pathogenesis. Mutations in genes such as SOD1 and CHCHD10 have been associated with mitochondrial abnormalities, including impaired mitochondrial function, abnormal mitochondrial morphology, and defects in mitochondrial dynamics [67, 68]. In multiple sclerosis (MS), mitochondrial dysfunction contributes to the sustained inflammatory phase of the disease, leading to energy imbalance and progressive neurodegeneration [69]. Across these diseases, mitochondrial dysfunction manifests as impaired energy production, oxidative stress, and altered calcium homeostasis, which are believed to be key factors in disease progression and severity.

## Glutamatergic Overstimulation: Unraveling the Role of Mitochondrial Deterioration

Glutamate is one of the major excitatory neurotransmitters in the nervous system. Glutamate serves a complex role by being both an amino acid as well as a neurotransmitter. It also performs a large array of physiological functions such as, chemical messenger, energy source for brain cells, regulation of learning and memory, pain transmission and mood regulation [70-72]. In a healthy brain, glutamate is tightly regulated; however glutamate dysfunction has profound effects both in disease and injury [73]. Notably, glutamate does not directly cause the neuronal death; rather, it initiates a cascade of neurotoxic processes, including cationic influx, mitochondrial dysfunction, energetic depletion and oxidative stress by the activation of glutamate receptors [70, 74, 75]. Both ionotropic (iGluR) and metabotropic (mGluR) glutamate receptors (GPCRs) are involved in glutamatergic neurotransmission. iGluRs are ligand-gated ion channels that are permeable to a variety of cations, including sodium, potassium, and calcium, whereas mGluRs are G-protein coupled receptors [71]. iGluRs are classified as kainite (mediate sodium influx), α-amino-3-hydroxy-5-methyl-4-isoxazole propionic acid (AMPA, mediate sodium influx), or N-methyl-D aspartic acid (NMDA, high calcium conductivity). Due to its high permeability to calcium ions, NMDAR has been pinpointed to be the primary cause of glutamate-induced neurotoxicity [72].

Under central nervous system homeostasis, synaptic glutamatergic clearance is accomplished by diffusion as well as transporter uptake into nearby glial cells, particularly astrocytes, and this clearance is independent of enzymatic breakdown [71]. When no synaptic event takes place, the baseline concentration of synaptic glutamate ranges from 25 nM to 600 nM. At this baseline concentration, neither glutamate receptor activation nor interference with neuronal excitability occurs [76]. Glutamate receptors are only active and contribute to neuronal signaling during a synaptic event when the glutamate concentration reaches 1.1 mM [77]. Excitotoxicity occurs when the synaptic glutamate concentration rises above 1.1 mM [77]. Persistent calcium influx into neurons occurs when glutamate receptors, particularly NMDARs, are overly activated [78]. This excessive glutamate results in the influx of calcium ions into the cell, disrupting normal cellular functions and ultimately causing cell death [76, 77]. Consequently, scavenging extracellular calcium reduced excitotoxicity-induced neuronal degeneration; however, this effect was not observed when cations other than calcium were eliminated, signifying the role of calcium in excitotoxicity (Fig. 1) [79, 80]. Due to an avalanche of calcium influx into neurons, mitochondria, an organelle responsible for bioenergetic homeostasis and controlling calcium signaling, is activated [81]. To maintain baseline levels of cytosolic calcium after excitotoxicity-induced calcium influx, mitochondria transiently capture cytosolic calcium and when required, gradually release calcium back into the cytosol [82]. One benefit of this process is that it momentarily buffers the intracellular calcium concentration, but it also eventually causes the mitochondrial membrane to depolarize, impairing the ability of mitochondria to combat oxidative stress as well as the production of ATP. As a result, neurons experience energy deprivation and produce high levels of ROS [83-87]. It has been discovered that calcium uptake by mitochondria is triggered when the cytosolic calcium concentration increases to 500 nM from baseline values of 50-100 nM [88]. The mitochondrial calcium uniporter (MCU) is a key player involved in mitochondrial calcium uptake. This uptake pathway is not activated until high calcium levels are spiked in the vicinity of the mitochondria, similar to what occurs during synaptic transmission [89]. MCU functions via several accessory proteins, including mitochondrial calcium uptake 1 (MICU1), MICU2, MICU3 (most dominant form in CNS) which forms a disulfide bond-mediated dimer with MICU1 but not with MICU2 and subsequently acts as an enhancer of MCU-dependent mitochondrial calcium uptake, mitochondrial calcium uniporter regulator 1 (MCUR1), essential MCU regulator (EMRE) and MCUb [90-94]. Several proteins found in mitochondria, such as leucine zipper EF-hand containing transmembrane protein 1 (LETM1) and the mitochondrial sodium calcium exchanger (NCLX), release calcium back into the cytosol [95, 96].

According to recent research, excitatory mitochondrial calcium dysregulation plays an adverse role in causing sub-lethal dendritic shrinkage, which is observed in chronic neurodegenerative disorders. Leucine-rich repeat kinase 2 is encoded by the LRRK2 gene, which is mutated in autosomal dominant PD and is implicated in excitatory mitochondrial toxicity. It was reported that when primary cortical neurons were transfected with either LRRK2-G2019S or LRRK2-R1441C, they displayed enhanced activity-dependent calcium influx through glutamate receptors and L-type calcium channels [97, 98]. Additionally, dendritic retraction occurs in postsynaptic regions, where a reduction in mitochondrial density is specifically observed [97, 98]. According to a study, fibroblasts from LRRK2-mutant patients presented higher levels of MCU and MICU1, which tend to increase the uptake of calcium by dendritic mitochondria after glutamatergic overstimulation [99]. Two recessive PD genes, PINK1 and Parkin, are involved in the management of calcium during glutamatergic excitotoxicity. Increased glutamine levels, an indirect indicator of glutamate neurotransmission, have been observed in Pink1 knockout rats [100]. PINK1 regulates the efflux of calcium from the mitochondria via the mitochondrial sodium/calcium exchanger [101]. Studies on PINK1 knockout in SH-SY5Y neuroblastoma cells have shown that excess mitochondrial calcium results from a malfunctioning sodium/calcium exchanger, which lowers the threshold for opening the mitochondrial permeability transition pore (mPTP), releasing cytochrome c, which triggers apoptosis [101]. Furthermore, PINK1-deficient cells exhibit an altered mitochondrial ultrastructure, with smaller, fragmented mitochondrial profiles with occasionally larger, swollen profiles and reduced cristae folds per µm of mitochondrial length [102]. Enhanced excitatory neurotransmission is also a result of the loss of Parkin (an E3 ubiquitin ligase). Parkin is known to be involved in pruning and degrading glutamatergic excitatory synapses [103]. Parkin is mutated in PD, which increases neuronal susceptibility to glutamate synaptic excitotoxicity. Parkin controls the crosstalk between the endoplasmic reticulum and mitochondria, which indirectly affects mitochondrial calcium levels [104]. It is believed that glutamatergic excitotoxicity is a significant pathophysiological event in AD. By activating glutamate receptors, specifically NMDA and AMPA, which are linked to the cognitive impairments and neuronal loss observed in AD brains, Aβ oligomers have been shown to cause mitochondrial damage [105]. When rat cortical neurons were incubated with 5 µM Aβ, mitochondrial calcium levels increased and subsequently decreased in response to the NMDA antagonists MK801 and memantine [106]. The subsequent activation of calpains, which are calcium-dependent proteases, and caspases (cysteine proteases) caused by mitochondrial calcium overload due to mitochondrial dysregulation causes apoptosis through both caspase-dependent and caspase-independent mechanisms [107]. In contrast, a study revealed that in PS2.30H transgenic mice expressing the FAD-linked mutant human PS2-N141I, mitochondrial calcium uptake instead of an increase was reduced, and this change was associated with glutamate-induced excitotoxicity. This observation could be linked to energy-deprivation along with the reduced capacity to handle stress [108].

The hyperactivation of NMDA receptors and glutamate excitotoxicity are described as central components of neurodegeneration in HD and are caused by several pathological CAG repeats in the huntingtin gene [109]. In HD mouse models, extrasynaptic NMDA receptor function is enhanced by an increase in the number of extrasynaptic receptors and an increase in the extracellular glutamate concentration [110]. The hyperactivation of glutamate receptors and their associated signaling pathways disrupts calcium homeostasis and leads to mitochondrial dysfunction, which is associated with alterations in ATP synthesis and bioenergetics in HD [111]. Glutamate overstimulation and altered NMDA receptor activity also lead to other phenomena, such as mitochondrial depolarization, aberrant mitochondrial dynamics, abnormal calcium handling, mitophagy, oxidative stress, complications in mitochondrial biogenesis, and intensified mitochondrial-dependent apoptosis in HD [112-114]. In the neuronal mitochondria of an HD mouse model, glutamate excitotoxicity-induced calcium dysregulation causes mPTP opening, and cyclosporine A, a drug that inhibits mPTP opening, effectively restores mitochondrial calcium handling and mitochondrial functions [115]. When brain ischemia occurs, the release of K+ and glutamate are possible initiating factors that induce cell death by triggering the overactivation of glutamate receptors, including NMDARs [116]. In transient focal ischemia model in Sprague Dawley rats, neuronal death was decreased by inhibiting cystine/glutamate antiporters concomitant with the release of glutamate into extrasynaptic sites and the activation of extrasynaptic NMDA receptors, which are the main causes of neuronal death [117]. OGD of cultured hippocampal neurons as an in vitro model for ischemia, extrasynaptic NMDA receptor signaling was upregulated, thereby inducing the expression of Clca1, which is a putative calcium-activated chloride channel and serves as a part of the pro-death program [118]. Due to an increase in intracellular calcium, mitochondrial calcium is dysregulated, leading to permeabilization of the inner mitochondrial membrane and opening of the mPTP during OGD [119]. Similarly, in traumatic brain injury (TBI), an increase in extracellular glutamate levels, and altered synaptic plasticity, have been observed [119-122]. Notably, glutamatergic synaptic mitochondria were found to be more susceptible to damage in TBI than non-synaptic mitochondria in terms of oxidative damage, fragmentation, and malfunctioning electron transport chains [124, 125].

## Oxytosis/Ferroptosis and Mitochondrial Interactions: Iron, Lipids, and Oxidative Stress:

**Figure 2.**
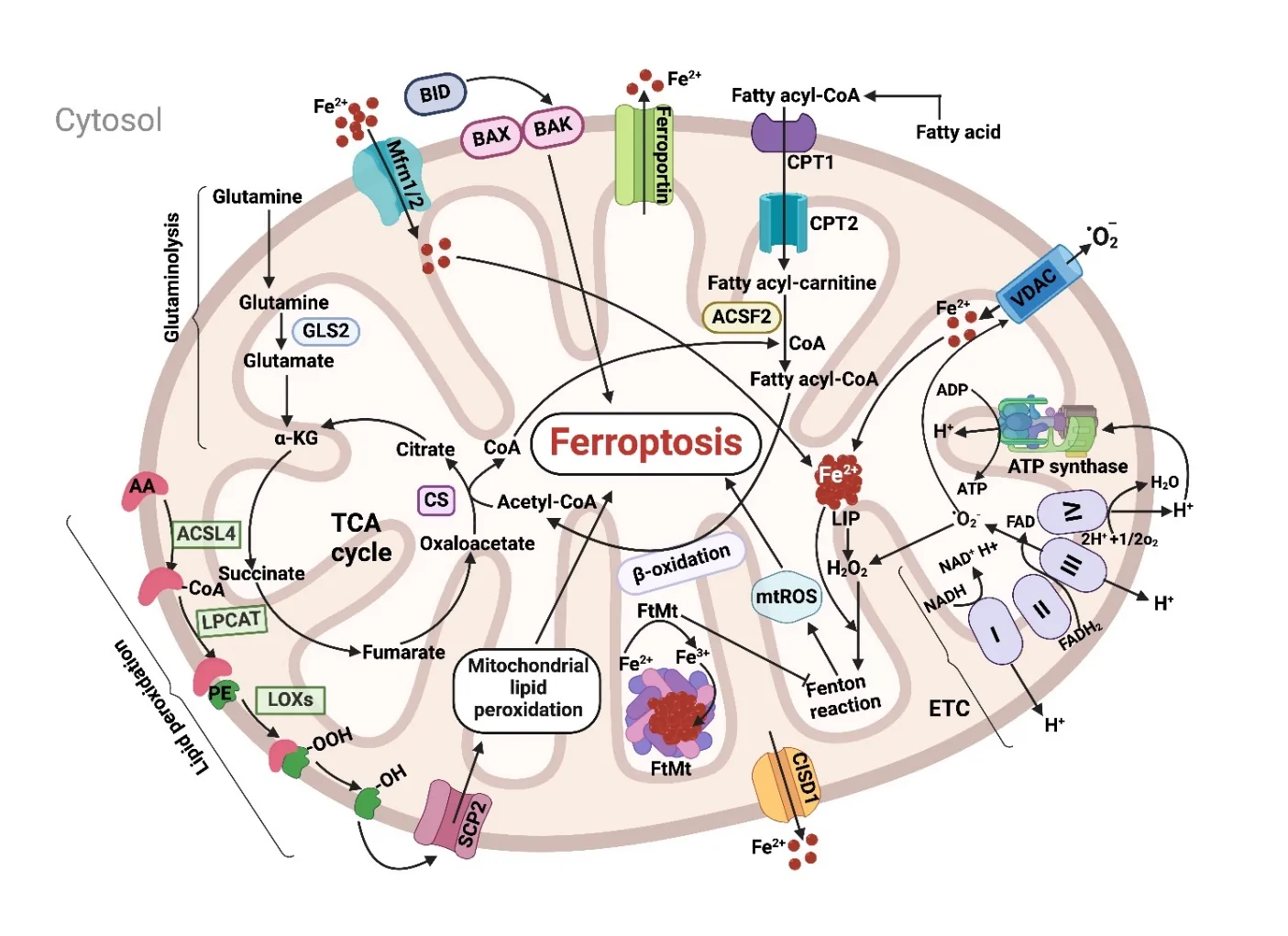
**Diagrammatic illustration showing the involvement of mitochondrial processes in ferroptosis**. Iron uptake via Mfrn1/2 increases the LIP concentration, promoting mitoROS generation through the Fenton reaction. BID triggers the activation of other proapoptotic proteins, such as BAX and BAK, contributing to ferroptosis. Fatty acids are first converted into fatty acyl-CoA and subsequently enter mitochondria via the carnitine shuttle system. In the matrix, they are again converted into fatty acyl-CoA via ACSF2, providing the specific lipid precursor for β-oxidation. VDAC imports Fe^2+^ into mitochondria. Fe^2+^ contributes to enhanced LIP, which in turn generates mitoROS through the Fenton reaction, thus promoting ferroptosis. CISD1 and ferroportin are involved in the export of mitochondrial iron, the decrease in the mitochondrial iron load and the suppression of ferroptosis. FtMt, by converting Fe^2+^ to Fe^3+^ through its ferroxidase activity and by storing Fe^3+,^ prevents the Fenton reaction. ACSL4, by converting arachidonic acid to arachidonoyl-CoA, provides a substrate for other enzymes, such as LPCA and LOXs, involved in lipid peroxidation, thereby driving ferroptosis. Glutamine in the cytosol is converted to glutamate by the mitochondrial isoform GLS2. Glutamate is converted to α-KG, thus providing fuel for the TCA cycle and lipid biosynthesis. CS regulates fatty acid synthesis through the release of CoA from acetyl-CoA, a precursor for β-oxidation, thus inducing ferroptosis. Abbreviations used: Mfrn1/2, mitoferrin 1/2; mitoROS, mitochondrial reactive oxygen species; LIP, labile iron pool; BID, BH3 interacting-domain death agonist; BAK, Bcl-2 homologous antagonist killer; BAX, Bcl-2-associated X protein (also known as bcl-2-like protein 4); ACSF2, acyl-CoA synthetase family member 2; VDAC2/3, voltage-dependent anion channels 2/3; ETC, electron transport chain; FtMt, mitochondrial ferritin; CISD1, CDGSH, Iron Sulfur Domain 1; ACSL4, long-chain-fatty-acid—CoA ligase 4; LPCAT, lysophosphatidylcholine acyltransferase; LOXs, lipoxygenase; AA, arachidonic acid; PE, phosphatidylethanolamine; CPT1/2, carnitine palmitoyltransferase 1/2; CoA, coenzyme A; GLS1/2, glutaminase 1/2; CS, citrate synthase; α-KG, alpha-ketoglutarate; TCA cycle: tricarboxylic acid cycle.

Oxytosis/ferroptosis is a unique non-apoptotic, controlled cell death process that recapitulates many aspects of mitochondrial impairment associated with neuronal cell death and has been linked to age-related neurodegenerative disorders [2, 3, 126]. Oxytosis/ferroptosis can be triggered by inhibiting the cystine/glutamate antiporter (system Xc-) with glutamate, which results in the loss of the endogenous antioxidant glutathione (GSH), excessive calcium influx into mitochondria, the production of ROS from mitochondria, lipid peroxidation across cellular membranes, and eventually cell death [3, 127]. Oxytosis/ferroptosis can also be induced by inhibiting glutathione peroxidase 4 (GPX4), a GSH-dependent antioxidant enzyme, via the use of RSL3 [126]. Systems Xc- and GPX4 are upstream and downstream targets of oxytosis/ ferroptosis, respectively, and both play important roles in this controlled cell death pathway [3]. The transplantation of functional exogenous mitochondria into both healthy and ferroptotic immortalized hippocampal HT-22 cells and primary cortical neurons was accompanied by enhanced metabolic activity and cell survival via the reduction of mitochondrial superoxide and lipid peroxidation, supporting the involvement of mitochondria in ferroptosis-induced cell death (Fig. 2) [128]. Additionally, the combined use of transcriptomic, proteomic and metabolomic approaches showed that oxytosis/ferroptosis induced by Aβ in MC65 nerve cells was strongly associated with impaired mitochondrial functions and metabolic reprogramming [129].

Mitochondria play a key role in cell metabolism during ferroptosis, including lipid and amino acid metabolism (involving cysteine and glutamine) [130]. Besides being the main architects of cell metabolism, glutaminolysis in mitochondria is a significant inducer of ferroptosis [131]. Glutaminolysis is an anaplerotic process in which glutamine is converted to α-ketoglutarate (α-KG) via glutaminase (GLS), which supplements tricarboxylic acid (TCA) cycle intermediates [132]. Citrate, produced by α-KG and its downstream TCA cycle metabolites, such as succinic acid, fumaric acid, and malic acid, plays a crucial role in initiating fatty acid metabolism throughout the cycle [131]. This shows that glutaminolysis contributes to the generation of lipid precursors needed for ferroptosis (Fig. 2) [131]. Glutamate produced by GLS from glutamine is an excitatory neurotransmitter, and synapses require low quantities of glutamate to function normally [133]. However, excessive glutamate binds to NMDAR in neurons resulting in oxidative toxicity and ferroptosis [133]. In contrast, in the absence of glutamine, ROS production, lipid peroxidation, and ferroptosis are suppressed [133]. Acetyl-CoA is a mitochondrial metabolite that is strongly reduced as a consequence of oxytosis/ferroptosis [129]. Acetyl-CoA is a key molecule that connects glycolysis to the TCA cycle, the metabolism of branched chain amino acids, and lipid synthesis/oxidation [129, 134]. Reduced levels of acetyl-CoA were found in oxytosis/ferroptosis-exposed HT22 nerve cells and in a mouse model of accelerated aging (SAMP8) [129, 135, 136]. Further, mitochondrial GLS 2 (not cytosolic GLS 1) catalyzes glutaminolysis during ferroptosis, suggesting the primary involvement of mitochondria in cell death [133]. Of note, ferroptosis induced by class 1 ferroptosis inhibitors is mitigated by glutaminolysis or glutamine starvation [126, 133]. However, supplementation of α-ketoglutarate and other TCA intermediates downstream of αKG restores ferroptosis under glutamine starvation conditions [137]. Further, inhibition of the αKG dehydrogenase complex blocks cystine starvation-induced ferroptosis [137]. In addition to glutaminolysis, mitochondrial fatty acid metabolism may provide the necessary lipid precursors for ferroptosis (Fig. 2). Knocking down acetyl-coenzyme A synthetase 2 (ACSF2) and citrate synthetase (CS), two enzymes required for mitochondrial fatty acid metabolism, has been shown to prevent erastin-induced ferroptosis [126]. These findings strengthen the role of mitochondrial fatty acid metabolism in ferroptosis [126].

Mitochondria not only provide the lipid precursors that are required for ferroptosis but also participate in the regulation of iron homeostasis in neurodegenerative diseases (Fig. 2) [138, 139]. Sustained iron exposure increased mtROS levels in dopaminergic neuroblastoma SH-SY5Y cells, while scavenging mtROS protected hippocampal neurons from iron overload damage by preserving mitochondrial membrane potential and morphological integrity [140, 141]. A recent study revealed that iron sequestration by mitochondrial ferritin (FtMt) was neuroprotective against oxidative stress and had a significant role in reducing neuronal damage under other conditions [142, 143]. Furthermore, FtMt overexpression dramatically reduced the cellular labile iron pool, inhibited H_2_O_2_-induced iron elevation, and protected cells from H_2_O_2_-induced damage [144]. FtMt is an important mitochondrial iron storage protein that has high homology with the heavy chain of cytosolic ferritin [145, 146]. FtMt has ferroxidase activity, which catalyzes the conversion of Fe^2+^ to the Fe^3+^ form for storage in the FtMt spherical shell [145]. Moreover, FtMt expression is tissue specific, with high levels found in cells with high oxygen consumption, such as those of the central nervous system and testes, but not in the spleen or liver, the main iron storage tissues [145]. These features indicate that the primary function of FtMt is to protect cells in certain organs against iron-dependent oxidative damage rather than being directly tied to cellular iron levels [145, 147]. Many studies have suggested that FtMt-deficient mice exhibit no obvious phenotypic or iron-related abnormalities under baseline feeding conditions but that FtMt has considerable protective effects under pathological conditions, such as in AD and PD [143, 147-149]. I/R-treated neuronal cells were reported to exhibit hallmark characteristics of ferroptosis, such as downregulation of GPX4, overexpression of PTGS2, and decreased mitochondria, and deletion of FtMt further exacerbates these changes [145]. Previous studies have shown that phosphatidylethanolamines increase the expression of PTGS2, a gene encoding cyclooxygenase-2 in cells experiencing ferroptosis, and PTGS2 upregulation is an appropriate downstream marker of ferroptosis [150-152]. Inhibition of FtMt also promotes the activation of microglia and inflammation, which in turn increases iron deposition in the brain through hepcidin-mediated inactivation of ferroportin1, which is a nonheme cellular iron exporter [145]. Many studies have reported that the expression of the ferroportin protein decreases with age in AD patients and in an AD mouse model [153-156]. Loss of ferroportin-associated increased iron accumulation in the brain enhances the expression and activity of the 12/15-lipoxygenase (12/15 LOX) enzyme, which catalyzes lipid peroxidation, ultimately leading to ferroptosis and cell death [153, 157, 158]. Furthermore, mitochondrial iron uptake is also regulated by mitoferrins 1 and 2, also referred to as SLC25A37 and SLC25A28, respectively, which are members of the mitochondrial carrier family [159]. Mitoferrin-1 and -2 (Mfrn1/2) boost mitochondrial iron uptake, which may be advantageous under normal conditions but can have adverse effects on iron dyshomeostasis, such as increased expression of Mfrn1/2 in PD models [160]. Aside from iron buildup, a defective iron regulatory mechanism is likely associated with mitochondrial dysfunction in HD patient brains, as evidenced by increased expression of mitoferrin 2 [161]. To investigate whether mitochondria are key sites of iron dysregulation in HD, Agrawal et al. discovered increased labile iron in the mitochondria of both mouse HD and human HD brains at an advanced stage, which was rescued by the chelating agent deferiprone, implying that mitochondrial labile iron overload may be an intermediary of HD mitochondrial dysfunction and disease progression [162]. Additionally, mitochondrial proteins such as iron-sulfur cluster assembly enzyme, cysteine desulfurase, CDGSH iron sulfur domain 1 (CISD1, also known as mitoNEET), iron-sulfur cluster assembly enzyme (ISCU), and CISD2 (also known as nutrient-deprivation autophagy factor-1 (NAF-1)) participate in the functional utilization of iron for iron-sulfur cluster biogenesis, acting to block ferroptosis by stimulating the biosynthesis of iron-sulfur clusters (Fe-S) and thus decreasing intracellular iron levels [163-166]. Genetic inhibition of CISD1 exacerbates iron-mediated intramitochondrial lipid peroxidation, which contributes to erastin-induced ferroptosis [163]. Pioglitazone a PPARγ agonist, on the other hand, reduces mitochondrial iron uptake, lipid peroxidation, and consequent ferroptosis by stabilizing the CISD1 iron sulfur cluster [163]. Mitochondrial iron accumulation is also found during ferroptosis related to Friedreich’s ataxia, and the mitochondrial-targeted antioxidant XJB-5-131 is effective at inhibiting ferroptosis [167].

During ferroptosis, the accumulation of lipids impairs mitochondrial functions and exacerbates mitochondrial dysfunction [168]. Mitochondrial lipid peroxidation is an important process in the development of ferroptosis in neurodegenerative diseases (Fig. 2) [169]. Using C11-BODIPY, a marker of lipid peroxidation, to monitor mitochondrial lipid peroxidation after sevoflurane exposure, Zhang and colleagues suggested a link between mitochondrial lipid peroxidation and ferroptosis [170]. Previous research revealed that iron ions (Fe^2+^) can enter mitochondria via VDAC1, resulting in iron buildup and cell injury [171]. VDAC1 expression is substantially increased in the brains of AD patients, AD transgenic animal models and Aβ_1-42_-induced AD-like pathology in PC12 cells [172, 173]. In neural cells, inhibiting VDAC1 was found to reduce glutamate-induced ferroptosis and mitochondrial fragmentation [174]. These data indicate that VDAC1 plays an important role in the progression of ferroptosis. Further, VDAC1 was shown to promote ferroptosis and mitochondrial dysfunction by inhibiting Wnt/*β*-catenin and adenosine monophosphate protein kinase (AMPK)/mTOR signaling *via* decreased GPX4 in Aβ_1-42_-induced PC-12 cells [172]. Reduced levels of GPX4 are a characteristic hallmark of ferroptosis [175]. Moreover, ferrostatin-1 (ferroptosis inhibitor), and silencing-VDAC1 increased the expression of GPX4 and ferritin heavy chain 1 and decreased Aβ-induced mitochondrial dysfunction, ROS accumulation, mitochondrial membrane potential, and disruption of mitochondrial respiratory chain complexes I and IV, further corroborating the incidence of ferroptosis in PC12 and SH-5HY cells [172]. Further, inhibiting the GSH/GPx4 pathway turns oxidative stress into a 12/15-LOX-dependent lipid peroxide signal, activating apoptosis-inducing factor (AIF) by inducing its translocation from mitochondria to the nucleus, and subsequently leading to neurodegeneration [176]. Moreover, the detrimental lipid signals from GPX4-/- cells were traced outside of the mitochondria, indicating that ferroptosis is triggered by lipid peroxidation outside the mitochondria [177]. Sterol carrier protein 2 (SCP-2) is a mitochondrial transporter that preferentially transports lipid peroxides to mitochondria, facilitating the transmission of oxidative stress within cells [178]. The inhibition of SCP-2 significantly protected mitochondria and decreased the levels of lipid peroxides and ferroptosis [179]. Similarly, inhibition of SCP-2 prevented erastin-induced ferroptosis, suggesting that mitochondrial lipid peroxidation is involved in this cell death pathway [177]. Studies using mitochondrion-targeted antioxidants have confirmed the impact of mitochondrial lipid peroxidation on ferroptosis. Nitroxide XJB-5-131, a mitochondrial-specific antioxidant, effectively prevents the generation of oxidized fatty acids from cardiolipin, a phospholipid found in mitochondria [131]. XJB-5-131 inhibits erastin- or RSL3-induced ferroptosis, indicating that protecting mitochondrial lipids may be adequate strategy to inhibit ferroptosis [131]. In addition, CISD1 prevents ferroptosis by inhibiting mitochondrial lipid peroxidation, thus confirming that mitochondrial lipid peroxidation promotes ferroptosis [163]. These findings validate the role of mitochondrial lipid peroxidation in ferroptosis.

In a study utilizing dopaminergic neurons, iron-dependent ferroptosis was reported to cause a decreased mitochondrial membrane potential, ATP generation, and increased mitochondrial ROS (Fig. 2) [180]. In sevoflurane-induced ferroptosis, the mitochondrial TCA cycle and ETC promoted ROS production, resulting in mitochondrial DNA damage [170] and amplified oxidative stress by triggering a vicious cycle of ROS production that damaged organelles, induced genomic instability and metabolic disequilibrium, and eventually neuronal apoptosis [170, 181, 182]. Staining with dichlorofluorescein and mitoSOX (a probe specific for mitochondrial ROS generation) in Aβ-treated MC65 cells, suggested that mtROS, a major hallmark of oxytosis/ferroptosis, leads to cell death [129]. Additionally, excessive mitochondrial fragmentation is associated with oxidative stress and cell death [182, 183]. In contrast, ferrostatin-1 decreased ferroptosis and mitigated mitochondrial dysfunction, as indicated by changes in the mitochondrial membrane potential, ATP production, and mtROS levels, indicating a link between these two conditions [172]. Furthermore, tert-butylhydroperoxide induces ferroptosis by activating the JNK1/2 and ERK1/2 signaling pathways, which act upstream of mitochondrial dysfunction, oxidative stress and ferroptosis in PC12 cells [185]. Consistent with these findings, a study suggested that JNK1/2 and p38 MAPK, but not ERK1/2, play important roles in erastin-induced ferroptosis in leukemia cells [186]. However, treatment with U0126, an ERK inhibitor, could reverse erastin-induced ferroptosis in HT1080 cells [126]. Moreover, RSL3, which mediates ferroptosis by lipid peroxidation and GPX4 knockdown in a glutathione-independent manner, impairs mitochondrial morphology and bioenergetics and increases mitochondrial fragmentation [187]. RSL3 altered mitochondrial biogenesis possibly through interfering with the AMPK/SIRT1/PGC-1α pathway, leading to extensive mtROS generation and ferroptosis [139]. PGC-1α responds to environmental and intracellular conditions for example temperature, physical activity and nutritional status, as well as increases the expression of respiratory genes in mitochondria and is regulated by SIRT1/3, TFAM, and AMPK, all of which play significant roles in mitochondrial biogenesis and function [188]. Additionally, matrine, which is an alkaloid found in herbal plants, has been shown to have a protective effect by upregulating the UCP2/SIRT3/PGC1α signaling pathway, which is helpful for attenuating ROS production and ferroptosis [189]. This study suggested the importance of mitochondrial biogenesis in the inhibition of ferroptosis and cell death [189]. Research using HT-22 neuronal cells has shown that ferroptosis-induced lipid peroxidation and increased ROS can activate the mitochondrial transactivation of the proapoptotic BCL2 family protein BH3 interacting-domain death (BID) agonist to mitochondria, which leads to severe alterations in mitochondrial function and integrity, suggesting a link between ferroptosis and mitochondrial dysfunction via a proapoptotic protein [190-193]. This leads to a decrease in the mitochondrial membrane potential and integrity, impaired ATP synthesis, increased ROS production, and the release of AIF from mitochondria [193, 194]. Similarly, cell death induced by deletion of GPX4 and associated lipid peroxidation involve mitochondrial release of AIF to the nucleus, where it mediates caspase-independent cell death [176]. Overall, the evidence suggests the substantial involvement of mitochondria in oxidative death, and targeting mitochondria is an essential therapeutic strategy for diseases featuring ferroptosis as an underlying mechanism of cell death.

## Dynamic Equilibrium: Mitochondrial Biogenesis and Quality Control through Biogenesis and Mitophagy

Ensuring the health of mitochondria and maintaining mitochondrial homeostasis require the critical process of mitochondrial quality control [195]. These pathways mainly involve mitochondrial biogenesis, mitophagy, fission and fusion, mitochondrial-derived vesicles and mitochondrial transport, all playing different roles. Promoting the generation of new mitochondria, clearing unwanted or damaged mitochondria, maintaining mitochondrial morphology via incessant fission and fusion, and degrading a portion of the mitochondrial content to prevent whole mitochondria from being damaged or transporting mitochondria to provide energy [196]. Fine tuning between mitochondria and the nucleus is required for mitochondrial biogenesis since only 13 proteins are encoded by mtDNA, and the remaining 1000 mtDNA proteins are encoded by the nucleus [197]. Crosstalk among various transcription factors occurs during the process of mitochondrial biogenesis. The PGC-1α-NRF1/NRF2-TFAM pathway is a crucial signaling pathway involved in mitochondrial biogenesis [198-200]. The master regulator of mitochondrial biogenesis, PGC-1α, acts as a transcriptional coactivator of several genes, including nuclear respiratory factor 1 (NRF1), NRF2, and TFAM, that increase the number of mitochondria as well as mtDNA [198-200].

**Figure 3.**
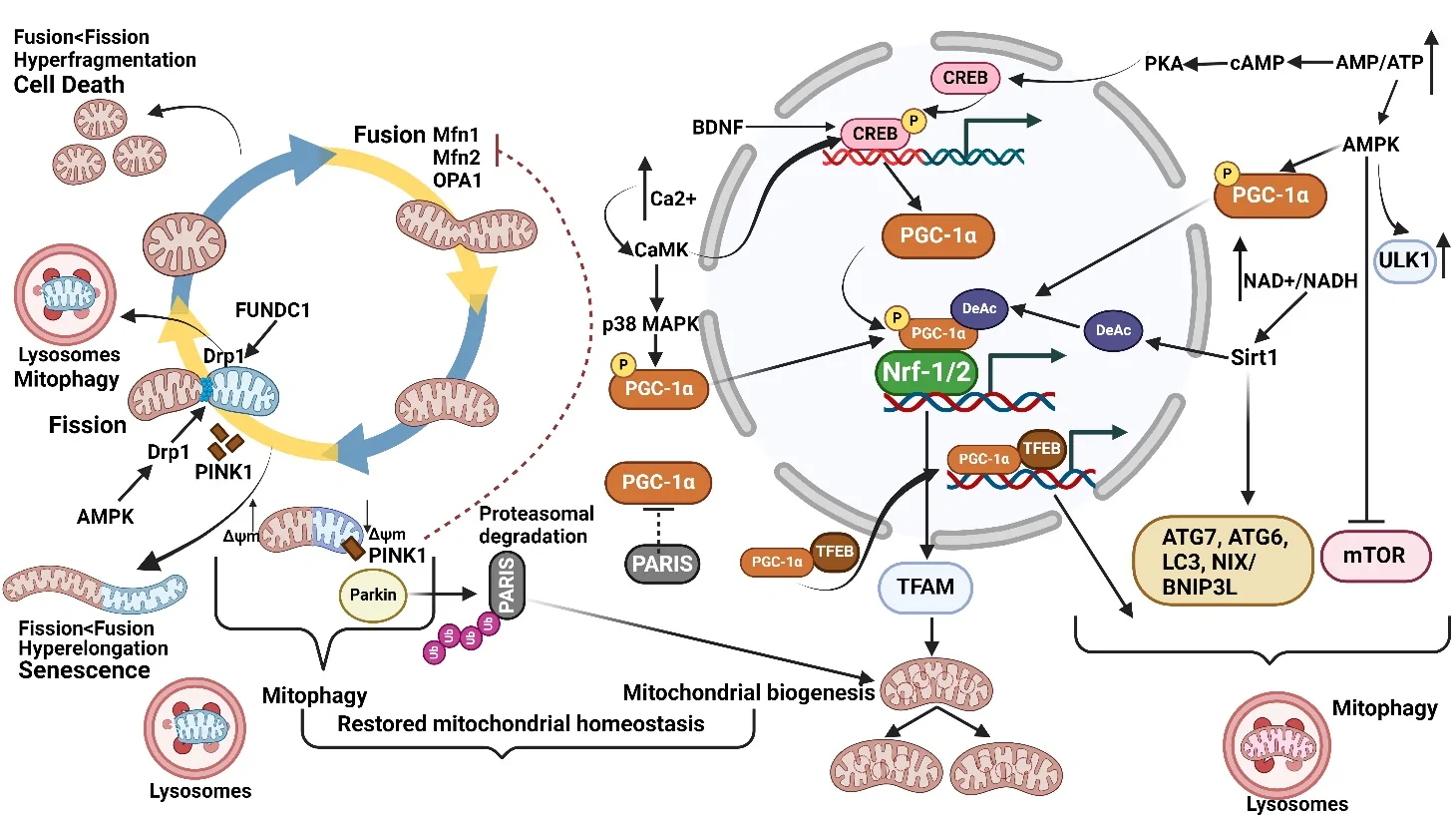
**Various signaling pathways involved in mitochondrial quality control, including pathways involved in mitochondrial biogenesis, mitochondrial dynamics through fission and fusion and mitochondrial autophagy (i.e., mitophagy), are involved**. Mitochondrial biogenesis is the process of increasing mitochondrial numbers and enhancing mtDNA transcription and translation. Under nutrient- and energy-deprived conditions, the ratio of AMP to ATP significantly increases, which activates cAMP, which in turn activates PKA. PKA is a kinase responsible for phosphorylating CREB. Phosphorylated CREB leads to the translation of PGC-1α, which is responsible for mitochondrial biogenesis. The AMP-to-ATP ratio also leads to the activation of AMPK, which phosphorylates PGC-1α and promotes its nuclear translocation. Similarly, increase in calcium levels also lead to the phosphorylation of PGC-1α and enhance its nuclear translocation via CaMK and p38MAPK. Sirtuins also promote the deacetylation of PGC-1α, thus leading to enhanced mitochondrial biogenesis via association with Nrf1/2 and leading to the synthesis of TFAM, which is responsible for mtDNA transcription and translation. BDNF also plays an important role in phosphorylating CREB and in turn increases the transcription of PGC-1α. A dynamic equilibrium is maintained between biogenesis and mitophagy, as these factors also play important roles in mitophagy. Sirtuins are known to activate various autophagy-related genes. AMPK leads to the increased synthesis of ULK1 and inhibits mTOR, and PGC-1α leads to the nuclear translocation of transcription factor EB (TFEB); these factors together promote mitophagy. In conditions of fission and fusion, Mfn1 and Mfn2 are required for GTP-mediated fusion of the outer mitochondrial membrane, and OPA1 is responsible for fusion of the inner mitochondrial membrane of two healthy mitochondria. Under conditions of neurodegeneration, the membrane potential of mitochondria decreases, which leads to mitochondrial dysfunction, and these mitochondria need to be removed to maintain homeostasis. For elimination, two processes are involved, mitophagy and other fission, to liberate the dysfunctional part of the membrane free from healthy mitochondria. Under conditions of low membrane potential, PINK1 accumulates on the mitochondrial membrane, which, in association with PARKIN, recruits mitophagy-promoting factors such as ATG7, ultimately leading to mitochondrial autophagy. Drp1 in association with PINK1 has been shown to promote mitochondrial fission. FUNDC1, an outer mitochondrial membrane protein, also facilitates Drp1-mediated fission of mitochondria. AMPK was also found to increase Drp1-mediated fission. Parkin interacting substrate (PARIS), which is inhibited by PGC-1α, is ubiquitinated by Parkin; thus, PGC-1α is no longer inhibited, and mitochondrial biogenesis occurs.

Several stimuli, such as the AMP/ATP ratio and calcium levels, play important roles in mitochondrial biogenesis [201]. When the energy status of the cell is compromised, the AMP/ATP ratio increases, and cells acquire signals for mitochondrial biogenesis to meet their energy demands [201]. Further, under stress, neurons become depolarized, with an increase in levels of AMP, activating AMPK, and subsequently phosphorylates PGC-1α, which in turn activates several mitochondrial biogenesis genes, including NRF-1/2 and TFAM [202, 203]. TFAM plays a role in mtDNA transcription and replication [204]. PGC-1α suppression via knockdown was reported to lead to a significant reduction in dendritic mitochondria, which in turn caused a decrease in the number of synapses (Fig. 3) [205]. Moreover, cAMP has been shown to act as an upstream regulator of mitochondrial biogenesis. Increased cAMP leads to the upregulation of protein kinase A-dependent activation of CREB, which in turn upregulates the expression of PGC-1α [206, 207]. Brain-derived neurotrophic factor (BDNF) is also involved in mitochondrial biogenesis in addition to conferring synaptic plasticity. According to a study, BDNF phosphorylates CREB, which increases PGC-1α, NRF1/2, and TFAM levels, which in turn increases mitochondrial biogenesis [205]. Additionally, BDNF-induced synaptic plasticity was repressed by knocking down PGC-1α [205]. Parkin also contributes to the activation of mitochondrial biogenesis by ubiquitination and UPS-dependent elimination of the transcription factor Parkin-interacting substrate (PARIS), which has a negative regulatory effect on PGC-1α and its target NRF1 (Fig. 3) [208]. Furthermore, in a study involving the dopaminergic neuroblastoma cell line SH-SY5Y, Parkin was found to directly associate with TFAM to increase the transcription of mtDNA-encoded genes [209]. Calcium ion has a very profound effect on the mitochondrial biogenesis with many studies reporting that increased calcium triggers the production of TFAM and PGC-1α responsible for mitochondrial biogenesis [210-212]. Further, inhibiting either CaMK or p38 MAPK reduces the effect of calcium on mitochondrial biogenesis [210-212]. Furthermore, CaMK activates PGC-1α via CREB [213]. These attributes to the fact that PGC-1α expression and activity are controlled by p38 MAPK and CaMK inhibitor prevented p38 activation in response to increase cytosolic calcium, thereby illustrating that p38 MAPK is a downstream target of CaMK [214]. The homeostatic balance between these pathways is crucial for maintaining mitochondrial health and energy homeostasis in the cells. Likewise, an increase in NAD+/NADH ratio also plays an important role in the biogenesis of mitochondria. In response to NAD+, Sirtuin 1 (Sirt1) deacetylates PGC-1α, thereby activating it, and PGC-1α has been found to upregulate mitochondrial density in axons [202, 215]. In several neurodegenerative diseases, such as AD, PD and HD, marked decreases in PGC-1α, NRF-1 and TFAM were found because mitochondrial biogenesis is disrupted during neurodegeneration [34, 216, 217].

Abnormal mitochondrial quality control and dysfunctional mitochondria are associated with several neurodegenerative diseases [52, 218, 219]; thus, a dynamic equilibrium is needed between mitochondrial biogenesis and mitophagy, which are central components of the mitochondrial quality control system for maintaining neuronal health. As neurons are highly energy intensive, it is necessary to maintain a healthy pool of mitochondria for both the maintenance of neuronal structural integrity and the ability to carry out specialized functions and for the preservation of neuronal health [220]. As one of the subtypes of macroautophagy, mitophagy specifically targets dysfunctional or damaged mitochondria to lysosomes for degradation to maintain homeostasis and facilitate the recycling of substances along with conservation of energy [221]. The cellular players that are involved in mitochondrial biogenesis are also involved in mitophagy, but only their mode of action becomes different. For example, PGC-1α, which is involved in mitochondrial replication, is also involved in mitophagy. PGC-1α stimulates the nuclear localization of transcription factor EB (TFEB), an essential mediator of autophagy and lysosomal biogenesis, thereby promoting mitophagy (Fig. 3) [222]. Through its interaction with the promoter, TFEB in turn increases the expression of PGC-1α. As a result, a vicious cycle between clearance and mitochondrial biogenesis maintains the steady state of mitochondria [223]. Similarly, AMPK, which was previously discussed to be involved in mitochondrial biogenesis, plays essential roles in mitophagy by phosphorylating the Ser317 and Ser777 sites on ULK1, thereby activating it and inhibiting mTOR by phosphorylating TSC2 and Raptor, which upregulates mitophagy under nutrient-deprived conditions [224]. Furthermore, sirtuins involved in mitochondrial biogenesis are also involved in mitophagy. SIRT1, a nuclear sirtuin that plays an important role in the upregulation of PGC-1α, also plays a crucial role in mitophagy since it activates a number of genes linked to autophagy and mitophagy, including ATG7, ATG6, LC3, and NIX/BNIP3L [225, 226]. Further, SIRT1 has been reported to be significantly downregulated in the parietal cortex in AD brains, and a negative correlation between SIRT1 and tau accumulation has been observed [227, 228]. Studies have also revealed that AD patients have lower levels of SIRT3, a mitochondrial sirtuin that is crucial for p62 clustering into ubiquitinated mitochondria and autolysosome formation [227, 228]. Similarly, cAMP, which is responsible for mitochondrial biogenesis, has been found to negatively regulate mitophagy, as its downstream effector PKA has been found to phosphorylate and inhibit LC3-II [229]. Parkin, as stated above, is involved in mitochondrial biogenesis and has also been shown to play an important role in mitophagy. According to previous research, mitochondrial malfunctions related to mitochondrial damage and depolarization prevents PINK1 internalization through mitochondrial translocase and promotes its accumulation on the outer mitochondrial membrane via the formation of PINK1-TOM-TIM23 super-complex assembly [230]. This assembly then recruits parkin to mitochondria, and both the E3 ubiquitinase and kinase activity of parkin lead to the formation of ubiquitin chains on substrates such as VDAC, FIS1, Mfn and TOM. These polyubiquitin chains are recognized by several autophagy adaptors, such as OPTN and NDP52, after they are phosphorylated by TANK-binding kinase 1 (TBK1), which in turn binds to autophagy-related ATG8 family proteins via the LIR motif, leading to mitophagy (Fig. 3) [231-239].

Mitochondrial fission and fusion are also subcomponents of the mitochondrial quality control process. Since mitochondria cannot be synthesized de novo, they originate from existing organelles by a continuous process of fission and fusion to maintain a healthy mitochondrial pool and regulate various cellular activities, such as growth, differentiation division and apoptosis [240]. The fusion of healthy mitochondria with one another is necessary to maintain the membrane potential, mtDNA replication, protein complementation, and distribution. This process is regulated via the mitofusin proteins (Mfn1 and Mfn2) on the OMM and by OPA1 on the mitochondrial inner membrane (IMM) [241]. At the OMM, GTP-mediated fusion of adjacent mitochondrial membranes is made possible via interactions between Mfn1 and Mfn2 [242]. Notably, insufficient mitochondrial fusion in the placenta causes Mfn1 and Mfn2 single knockout mice to die before birth and double knockout mice to die in the middle of gestation, emphasizing the role of these proteins in mitochondrial dynamics [243]. OPA1, which is present on the IMM, is necessary for effective fusion of the IMM. OPA1-mediated mitochondrial fusion requires both membrane-bound OPA1 and short-soluble OPA1, which are cleaved at the S2 site by the Yme1L protease [243-245]. Mitochondrial fusion promotes overall mitochondrial function by enabling calcium and ROS buffering as well as the exchange of mitochondrial contents (Fig. 3) [246]. Although mitochondrial fusion does not directly cause mitochondrial biogenesis, it is essential for preserving the integrity and health of mitochondria, which in turn helps the biogenesis process [247, 248]. PKA, the downstream effector of cAMP, has been found to dephosphorylate Drp1, sequestering it in the cytoplasm, leading to fusion and preventing unnecessary autophagic degradation of elongated mitochondria [249]. Moreover, PKA plays an important role in mitochondrial biogenesis, as stated above. The morphology, distribution and function of mitochondria are regulated by the equilibrium between mitochondrial fusion and fission. Fusion and fission serve as a bridge between biogenesis and mitophagy. Mitophagy is prevented by mitochondrial fusion, whereas fission is a crucial prerequisite for mitophagy [247].

Mitochondrial fission controlled by Drp1 and its receptor proteins fission 1, mitochondrial fission factor, and mitochondrial dynamics proteins help in the removal of damaged mitochondria via mitophagy and prevents these organelles from rejoining healthy mitochondria [250]. There is a dynamic interaction between mitophagy-related proteins as well as between mitochondrial fission and fusion-related proteins. Research has shown that PINK1 can relieve the inhibitory effect of PKA on Drp1 and indirectly activate Drp1, triggering mitochondrial fission (Fig. 3) [251]. Mfn1 and Mfn2, pro-fusion mediators, are substrates of Parkin. Ubiquitination and subsequent degradation of Parkin restrict damaged mitochondria from entering the healthy mitochondrial pool and fusing with healthy mitochondria before mitophagy [252]. Additionally, Mfn2 is a tether protein between the mitochondria and the ER. Parkin-dependent ubiquitination of Mfn2 disrupts the affinity between mitochondria and the ER, facilitating the availability of substrates on the MOM to PINK1 and Parkin and the subsequent induction of mitophagy [253]. AMPK is also linked to mitochondrial fission and mitophagy. Under stress, AMPK phosphorylates mitochondrial fission factor, a MOM receptor for Drp1, causing Drp1 to be recruited to the mitochondria. This process promotes mitochondrial fission and clearance by activating ULK1 [254]. FUN14 domain containing 1 (FUNDC1) is a MOM protein that is also involved in the process of mitochondrial fission and fusion, linking it with mitophagy [255, 256]. To control mitochondrial fission and fusion, FUNDC1 can interact with Drp1 and OPA1 through their cytosolic domain and K70 site, respectively [255, 256]. Under normal conditions, OPA1 is anchored by FUNDC1, thereby preventing its cleavage from L-OPA1 to S-OPA1, which facilitates mitochondrial fusion. Under conditions of mitochondrial stress, mitochondrial fission and mitophagy are promoted by an increase in the association of FUNDC1 with Drp1 and a decrease in the interaction of FUNDC1 with OPA1 (Fig. 3) [255, 256].

These findings suggest that impaired mitochondrial quality control, in terms of reduced biogenesis and abnormal mitophagy, can contribute to the pathogenesis of several neurodegenerative disorders, including AD, PD, HD and ALS [34, 257, 258]. It has been documented that in neurodegenerative diseases, there is an increase in the rate of mitochondrial fission, which increases the susceptibility of neurons to cell death [259-261]. Thus, maintenance of mitochondrial integrity and functionality is of utmost importance to brain energy generation, and a dynamic equilibrium should exist between biogenesis, mitophagy and fission or fusion to maintain mitochondrial homeostasis. For this coordination to occur, harmony should be maintained between several mediators and transcription factors involved in mitochondrial quality control. Targeting mitochondrial quality control to preserve and restore mitochondrial function may serve as a promising therapeutic avenue for targeting and mitigating neurodegenerative diseases.

## Oxidative Stress in Neurodegenerative Cascades: Intersection of Excitotoxicity and Mitochondrial Dysfunction

The prevalence of neurodegenerative disorders is increasing globally in parallel to the aging population. Although the molecular pathways underlying neurodegeneration are unclear, the central nervous system is especially susceptible to oxidative damage due to excess ROS, which can modify membrane permeability, induce damage to the respiratory chain of mitochondria, cause mutations in mitochondrial DNA and affect the homeostasis of calcium and the defense mechanisms of mitochondria. All of these alterations are linked to the onset of various neurodegenerative disorders by exacerbating or mediating neuronal dysfunction and initiating neurodegeneration. Excitotoxicity, described as damage and eventual death of neurons caused by prolonged or excessive exposure to excitatory amino acids, has also been demonstrated to play a crucial role. Excessive glutamate release and/or reduced glutamate uptake lead to disruption of calcium homeostasis in neurons, which in turn causes oxidative stress, mitochondrial dysfunction, abnormalities in protein turnover and neuroinflammation [262]. Because oxidative phosphorylation in mitochondria is the main contributor to the production of ROS, there is an inherent connection between oxidative stress and anomalies in mitochondria in neurodegenerative disorders [63]. The inner mitochondrial membrane has a variety of free radical scavengers and enzymatic ROS clearance mechanisms for protection under normal physiological conditions. However, it is evident that mitochondrial defense mechanisms could be weakened under some pathological conditions as a result of either genetic mutations or a spike in the production of free radicals [263]. However, it is typically challenging to determine whether mitochondrial abnormalities are the underlying cause of toxicity or secondary collateral damage. However, a myriad of studies appears to suggest that oxidative stress resulting from mitochondrial dysfunction is the principal mechanism linked to neurodegeneration [264, 265]. Furthermore, there is growing recognition of the notion that the respiratory chain can sustain self-inflicted damage from mtROS generation, leading to a cycle of additional damage to mitochondrial proteins and increased production of ROS [266]. Furthermore, the low ability of mtDNA to repair itself and the absence of protective histones make mtDNA an accessible target for ROS [267]. The most convincing evidence regarding the involvement of mitochondrial abnormalities and consequent oxidative stress is particularly evident in AD and PD, both of which are superimposed on inherited factors and most likely contribute significantly to the spontaneously occurring forms of these two disorders [268-270]. The oxidative damage to polyunsaturated lipids, proteins and DNA/RNA observed in both AD and PD patients is believed to be caused at least in part by an excess of redox-active metals [271-273]. In AD, ROS/RNS-induced oxidative stress impacts the mitochondrial membrane potential as well as neuronal glutamate and glucose transport [274]. Furthermore, it affects sodium-potassium ATPase activity, which is the pump responsible for generating action potentials and disrupting neuronal calcium homeostasis, the factors that lead to the impairment and degeneration of neurons [275]. It is quite plausible that Aβ can cause oxidative stress, most likely because of the complexes it forms with redox-active metals. Binding of zinc, copper and iron to Aβ facilitates its aggregation into plaques [276]. It has also been revealed that among these metals, copper forms the most stable complex with Aβ, leading to the eventual production of hydrogen peroxide and superoxide [277]. The resultant oxidative damage induced by these metal–amyloid complexes has been shown to be responsible for excitotoxicity, thereby promoting depolarization of the membrane and compromising mitochondrial function [278]. Mitochondrial malfunction, as previously mentioned, exacerbates oxidative stress, perpetuating a vicious cycle. The pathogenesis of PD is primarily driven by the degeneration of dopaminergic neurons in the substantia nigra pars compacta, which results in the typical motor symptoms linked to the condition [279]. There is consistent evidence linking mitochondrial malfunction and oxidative stress to the cascade of events underlying the degeneration of dopaminergic neurons [280, 281]. Increased levels of oxidative stress indicators such as 4-hydroxyl-2-nonenal (4-HNE), 8-hydroxy-guanosine and 8-hydroxy-deoxyguanosine (8-OHDG) have been frequently observed in postmortem examinations of PD brains [282]. The finding that brain regions damaged by PD possess abnormally elevated amounts of redox-active metals, especially iron, is consistent with the evidence of oxidative stress in this disorder [283, 284]. α-Synuclein aggregates, a pathogenic feature of PD, have also been linked to mitochondrial dysfunction and elevated oxidative stress [285]. Oxidative stress and mitochondrial dysfunction are also frequently involved in the death of neurons linked to other neurodegenerative conditions, such as amyotrophic lateral sclerosis (ALS), multiple sclerosis (MS), HD, stroke-related brain and spinal cord damage, and TBI [286-288]. Impairment in protein aggregation, folding and trafficking, inflammation, glutamate excitotoxicity, mitochondrial dysfunction and oxidative stress are among the biochemical pathways that are thought to be responsible for neurodegeneration in individuals with ALS [289]. Enhanced vulnerability to oxidative stress and impaired mitochondrial function have been linked to the aggregation of misfolded superoxide dismutase type-1 (SOD1) in mitochondria in the spinal cords of SOD1G37R mice and SOD1G93A rats [290]. Moreover, aberrant interactions between mutant SOD1 and mitochondria are mediated by oxidized cysteine residues in motor neuronal NSC-34 cells in mice, resulting in a disturbance in the redox balance of the mitochondria and a consequent increase in oxidative stress [291]. The preferential binding of aberrant proteins to mitochondrial ribosomal proteins increases ROS generation and mitochondrial membrane potential, which in turn stimulates oxidative stress-associated damage in ALS [292]. Numerous investigations have demonstrated that oxidative stress plays a significant role in the pathogenesis of HD [293-295]. HD is associated with increased levels of oxidative stress markers, such as 8-OHDG, protein carbonylation products, 4-HNE, 3-nitrotyrosine and isoprostanes [294, 296-298]. Cellular DNA repair mechanisms are hampered by mutant huntingtin, which impedes the functions of the ku70 DNA repair protein, thus facilitating unregulated DNA damage [299]. Furthermore, mutant huntingtin elevates neuronal ROS levels [300]. Thus, unregulated DNA damage combined with oxidative stress may result in the death of neurons in HD. Additionally, mutant huntingtin has been demonstrated to cause cytotoxicity primarily through the kynurenine pathway, with the subsequent generation of ROS and quinolinic acid, both of which promote mitochondrial malfunction, oxidative stress, and neuronal degeneration [301]. Alterations in mitochondrial calcium homeostasis and DNA damage triggered by oxidative stress have also been suggested to be significant mechanisms through which mitochondrial malfunction results in neuronal degeneration in HD [302, 303]. Together, the evidence of mitochondrial malfunction and an excitotoxic component apart from inflammation likely indicates the mechanism underlying neuronal degeneration in MS patients. Neurotoxicity of mitochondrial proteins in MS patients leads to impairment of the mitochondrial membrane potential and apoptosis of cells [304]. Iron and other metal levels are elevated in MS, particularly in the area surrounding lesions. It has also been reported that impaired metabolism of iron and iron-mediated oxidative stress play essential roles in the etiology of MS [305]. Additionally, excitotoxicity plays a crucial role in the neuronal impairment that results in apoptotic cell death in MS [306]. Studies demonstrating elevated levels of glutamate transporters in MS patients suggest that glutamate excitotoxicity might play a role in the pathogenesis of this disorder [307]. Furthermore, a significant intrinsic reservoir of free radicals during ischemia is the mitochondrial matrix. During cerebral ischemia, increased generation of ROS, reduced synthesis of ATP and the secretion of proapoptotic signals can all result from oxidative damage to mitochondria, triggering the process of mitochondrial necrosis and resulting in cellular degeneration [308, 309]. Moreover, in the damaged brain or spinal cord, mitochondrial oxidative stress and subsequent mitochondrial malfunction play particularly significant roles in the cascade of posttraumatic cellular death [310-312]. An intracellular buildup of calcium ions that occurs quickly after injury is the cause of this mitochondrial homeostatic failure. This prompts the mitochondria to attempt buffering (sequestering) excessive calcium ions, which ultimately results in reduced oxidative phosphorylation and mitochondrial respiratory failure [311, 312]. A primary driver of this cascade is excitotoxicity, which triggers a variety of processes, including the binding of NMDA, depolarization and increased ion flux (especially calcium); in turn, this cascade promotes the production of ROS from mitochondria and acidosis along with immunopathology, especially the release of ROS from neutrophils [313]. Thus, neurodegeneration tends to be consistently associated with imbalances in pro-oxidant/free radical levels and antioxidant defensive mechanisms, with the system frequently predisposed toward excessive pro-oxidants, excitotoxicity and mitochondrial dysfunction.

## Clinical Trials targeting Mitochondrial Dynamics in Neurodegenerative diseases

While various therapies aim to target mitochondria, the use of small molecules and peptides has emerged as a promising modality owing to their ease of preparation and administration, cost-effectiveness, low molecular weight, and high bioavailability. Many clinical trials are being held to prevent neurodegenerative diseases with much focus on introducing antioxidants to prevent ROS from damaging mitochondria and other cellular structures [314]. Out of the 32 clinical trials for AD listed in Supplementary Table 1, nicotinamide riboside in the form of dietary supplements has gained all the attention. Nicotinamide functions by lowering the levels of Thr231-Phosphotau, inhibiting sirtuin, and converting itself to NAD+ [315]. Nicotinamide and MIB-626 (a nicotinamide precursor) (NCT05040321) are potential therapeutics with several ongoing clinical trials listed in Supplementary Table 1. It was discovered that hydralazine, a drug approved by FDA for hypertension, was helpful in preventing or delaying oxidative stress related diseases such as AD [316]. This drug works by activating the nuclear factor erythroid-derived 2-related factor (Nrf2) pathway, leading to transcription of antioxidant genes and prevention of oxidative stress [316]. Hydralazine is also under successful clinical trial for mitigating AD (NCT04842552). A clinical trial (NCT05591027) is being conducted on an extract of *Centella asiatica*, which is also known to activate the Nrf2 pathway [317]. Since, impaired insulin metabolism in the AD brain [318], therefore, intranasal insulin delivery in combination with empagliflozin (FDA-approved Type 2 Diabetes drug), is being evaluated clinically (NCT05081219) for mitigating AD and to enhances cognitive and memory abilities [318]. Apart from the conventional therapeutics, transcranial photobiomodulation is the non-drug AD therapy undergoing clinical studies (NCT04018092). This technique employs the usage of electromagnetic radiation in the visible and near-infrared ranges to activate enzymes such as NADH dehydrogenase, cytochrome c oxidase, and cytochrome c reductase [319]. It also reduces amyloid beta in vitro [320]. However, several clinical trials are withdrawn or terminated due to several reasons. For example, the clinical trial of MitoQ dietary supplement (NCT03514875) was withdrawn due to change in operating plans prior to study initiation and enrolment. Another clinical trial (NCT03702816) was terminated concerning GE180 PET Scan in patients with AD and PD, because GE180 has limited Blood-brain barrier permeability reducing its efficacy (Supplementary Table 1).

For PD out of 17 clinical trials listed in Supplementary Table 1, the trials which are widely popular include nicotinamide riboside supplementation (NCT05344404, NCT03568968). Interestingly, nicotinamide riboside is being evaluated in clinical trials for PD and AD, indicating the significance and effectiveness of this treatment for these neurodegenerative diseases. Further, a Phase 2 clinical trial of Ursodeoxycholic Acid (UDCA) (NCT03840005) in PD patients have also shown reliable results in terms of small but significant improvements in motor abilities over a year along with improved mitochondrial functions [321]. Similarly, a phase 2 clinical trial of MitoQ (NCT00329056) for treatment of PD have shown reduction in Unified Parkinson's Disease Rating Scale (UPDRS) score, indicating improvement in PD symptoms. Furthermore, clinical trial study on Vitamin D supplementation in PD patients (NCT04768023) have shown significant decrease in the concentration of the inflammatory marker TNF-α after vitamin D3 administration in PD patients with Deep brain stimulation. Additionally, the neuroprotective metabolite Kynurenic acid was found to be higher in vitamin D treated group [322]. Even though there have been positive results from a number of PD clinical trials, many have failed. One such trial is creatine supplementation, which has shown neuroprotective effects in mouse models [323]. However, its effectiveness in PD patients is still debated, as it had little to no effect on the Unified Parkinson's Disease Rating Scale scores [323, 324]. Another proposed therapy is coenzyme Q10 administration, which forms ubiquinol, an antioxidant found in mitochondrial membranes [325]. Although there is ongoing discussion on the effectiveness of coenzyme Q10 therapy in PD patients, coenzyme Q10 treatment has protective effects on DA neurons in a mouse model of PD [323, 326]. Melatonin, another clinical trial (NCT04287543), was withdrawn due to global lockdown during the COVID-19 pandemic, highlighting the failure of clinical trials due to geographical conditions.

For HD, the number of clinical trials is very low owing to its rare nature. It is a type of neurodegenerative disease that slowly progress over decades. Therefore, it is very challenging to detect changes in disease progression during a relatively short-term clinical trial. Out of 4 mentioned clinical trials in Supplementary Table 1, one clinical trial involving creatine monohydrate supplementation (NCT00712426) was terminated because of the ineffectiveness of creatine in slowing down the loss of function in early symptomatic HD.

## Therapeutic Candidates Targeting Mitochondrial Bioenergetics

A decrease in brain bioenergetics resulting from mitochondrial dysfunction in neurodegenerative diseases serves as a prominent biomarker of disease presence even before symptom onset [327]. This phenomenon can be targeted by pharmacological strategies, focusing on drug candidates capable of affecting the electron transport chain (ETC) and oxidative phosphorylation (OXPHOS) in the treatment of neurodegenerative diseases [328]. A recent review study collected data on mitochondrial ETC complexes involved in OXPHOS, suggesting their potential as targets for small molecule therapeutics to enhance mitochondrial activity and cellular energy [329]. The use of small molecules such as metformin, resveratrol, berberine, and epigallocatechin-3-gallate (EGCG) to partially inhibit mitochondrial complex I (CI) has been identified as a potential treatment for conditions such as cancer and neurodegenerative disorders [329]. J147, a derivative of curcumin, has been explored for treating AD by preventing or slowing neurodegeneration [330]. It has been shown to improve memory, restore cognition, and maintain synaptic protein levels in mouse models. J147 safeguards the brain from age-related toxicity by specifically engaging with the α-F1 subunit of ATP synthase. This targeting action leads to modulation of the AMPK/mTOR pathway, contributing to the overall protective effects on the brain associated with aging [331]. Coenzyme Q10 (ubiquinone; CoQ10) and its derivatives have been extensively studied as investigational products. Ongoing efforts involve the development of mitochondrial-targeted analogs of CoQ10 to promote oxidative phosphorylation (OXPHOS) and reduce oxidative damage. Several studies have provided evidence for the neuroprotective potential of CoQ10 in AD, as indicated by its ability to decrease Aβ plaque deposition and alleviate memory deficits in vivo [332]. However, despite these promising preclinical findings, clinical outcomes presently do not favor the use of CoQ10 or its analogs for treating AD [333]. Among the studied mitochondrial-targeted CoQ analogs, mitoquinol (MitoQ) and plastoquinonyl-decyl-triphenylphosphonium (SkQ1) have been investigated for their bioenergetic effects. MitoQ, which is localized in mitochondria, mitigates AD symptoms, including synaptic loss, Aβ accumulation, and oxidative stress, in the 3xTg-AD model [334]. Additionally, at nanomolar concentrations, SkQ1 exerts positive effects by mitigating age-related decreases in and decreasing the pathological buildup of Aβ and the hyperphosphorylation of Tau in an OXYS rat model. This model is regarded as a suitable representation of sporadic AD [335]. Various other triphenylphosphonium-conjugated antioxidants, such as MitoVitE, MitoLip, MitoTempol, and MitoPrx, are being explored, and efforts are underway to develop additional antioxidants, such as MitoCurcumin [336]. Like MitoQ, MitoVitE, also known as mitotocopherol, is efficiently taken up and accumulates in mitochondria. It also has protective effects against hydrogen peroxide, lipid peroxidation, and oxidative stress in various tissues, including the brain. In fibroblasts from Friedreich's ataxia patients, MitoVitE has been shown to prevent cell death induced by excessive ROS production [337]. Additionally, in bovine aortic epithelial cells, MitoVitE inhibits the release of cytochrome c at a concentration of 1 mM, preventing apoptosis while restoring proper bioenergetics. Another study highlighted its effectiveness in combating neurodegeneration in cerebellar granule cells. This is achieved by diminishing the buildup of intracellular oxidants stimulated by ethanol [338].

While animal studies on PD have shown promising results with certain compounds, clinical trials face challenges, and novel formulations or derivatives of CoQ10 (e.g., EPI 589) are being developed [339]. Vitamin K2, specifically long-chain menaquinone 7 (MK-7), has shown structural similarity to coenzyme Q10 (CoQ10), as evidenced by studies in Drosophila flies carrying a homozygous PINK1 knockout [340]. However, it is essential to note that these findings require validation through human trials. Nicotinamide and its analogs are currently being explored for their ability to normalize redox levels. Nicotinamide may also impact sirtuins. Nicotinamide, which is particularly relevant for CI and potentially beneficial for electron transport chain (ETC) disturbances, involves NAD^+^ and NADH (NCT03815 916) [341]. An investigational drug candidate evaluated in vivo is N-methyl, N-propynyl-2-phenylethylamine (MPPE), which functions as a MAO-B inhibitor. It hinders the MPTP-induced loss of nigral cells, enhances mitochondrial superoxide dismutase activity to decrease oxidative stress, and enhances the function of mitochondrial CI [342]. Acetyl-L-carnitine (ALC) is an inherent constituent of the mitochondrial inner membrane (MIM) that has the capacity to traverse the BBB easily and provide acetyl groups facilitating acetyl-CoA synthesis. Acetyl-CoA is crucial for the tricarboxylic acid cycle and the generation of NADH and FADH2, which supply electrons to the electron transport chain (ETC), thereby circumventing the requirement for pyruvate dehydrogenase (PDH). Additionally, ALC increases glutathione production [343]. Additionally, (S)-equol enhances mitochondrial respiration and glycolytic flux in hippocampal neurons. Moreover, it amplifies COX1 activity and expression in the brains of mice subjected to ovariectomy [344]. Ursodeoxycholic acid (UDCA), a clinical candidate commonly used in chronic inflammatory liver disease and known for its extensive safety profile, has shown promise [345]. UDCA has been proven to inhibit mitochondrial membrane depolarization and maintain the stability of cytochrome c within the mitochondrial membrane [346]. Compelling in vivo data support the notion that UDCA could offer specific advantages in addressing mitochondrial dysfunction among individuals with the LRRK2^G2019S^ mutation [347].

**Figure 4.**
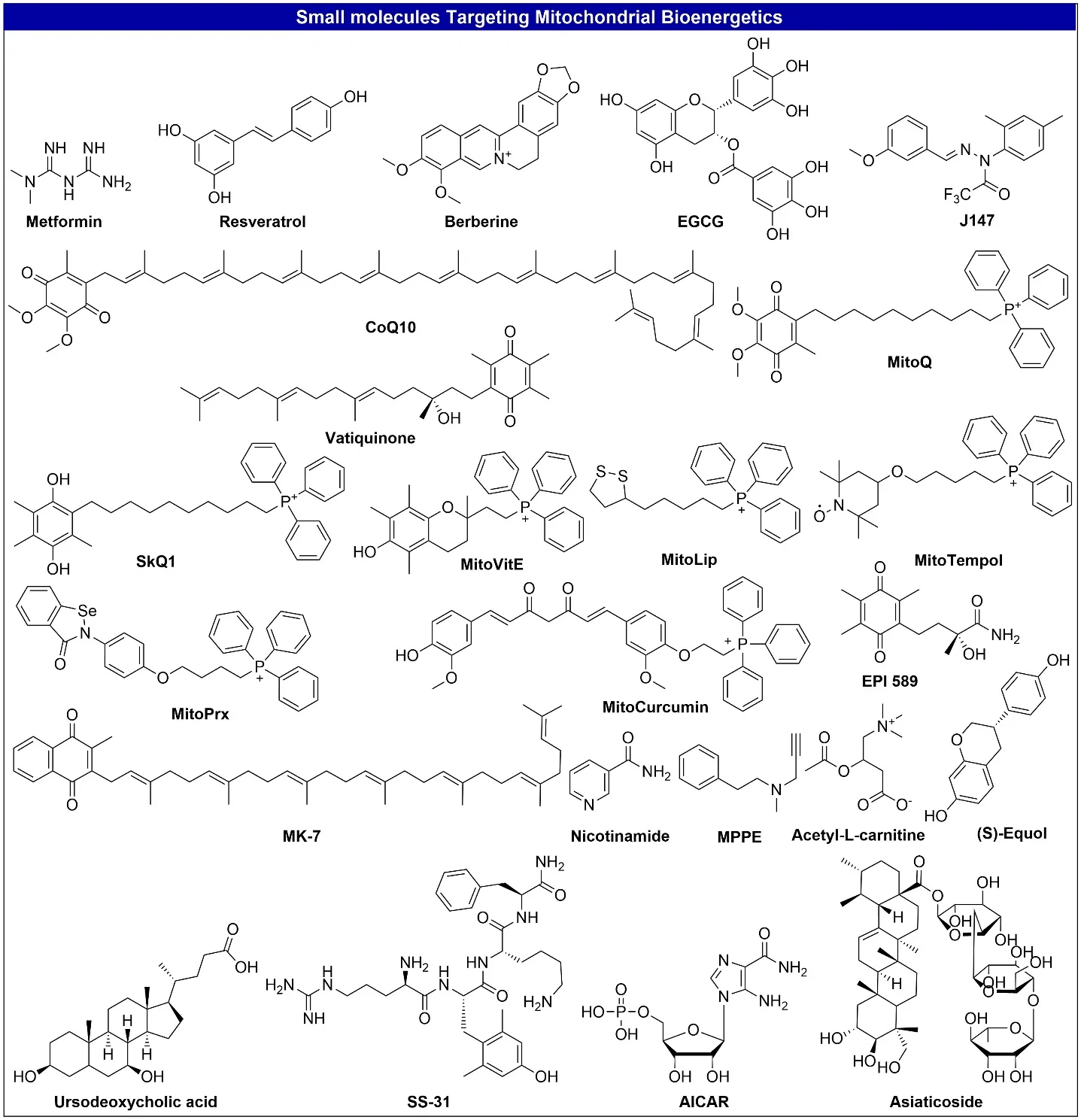
Chemical structure of therapeutic candidates targeting mitochondrial bioenergetics.

A clinical trial evaluating oral UDCA and exploring brain energy metabolism using 1H-MRSI (NCT02967250). SS-31 (H-D-Arg-Dmt-Lys-Phe-NH_2_), an opioid agonist peptide, enhances ATP production by binding to cardiolipin, a crucial phospholipid for the activity of proteins within the mitochondrial inner membrane (MMI) [333]. SS-31 demonstrates efficacy in ameliorating mitochondrial dysfunction and oxidative stress in primary neurons from mice expressing amyloid precursor protein (APP). In vivo, the intraperitoneal administration of SS-31 over a six-week period suppressed Aβ production and maintained mitochondrial function as well as synaptic activity in mice harboring the APP gene [347]. A study conducted on G93A transgenic mice, which serve as models for amyotrophic lateral sclerosis (ALS), demonstrated that the administration of SS-31 resulted in a decrease in oxidative stress markers and prevented neurodegeneration [348]. Moreover, considering that AMPK plays a role in enhancing energy metabolism by upregulating processes such as glucose uptake, glycolysis, and mitochondrial biogenesis, the specific activator of AMPK, 5-aminoimidazole-4-carboxamide ribonucleotide (AICAR), has been demonstrated to inhibit amyloid-beta (Aβ) secretion from primary neurons in mice with AD [349]. This inhibitory effect is counteracted by an inhibitor of AMPK. These results suggest a mechanistic link between the origin of AD and compromised metabolic signaling. This finding implies that interventions aimed at activating pathways that regulate bioenergetic function and glucose metabolism in the brain could serve as promising therapeutic approaches for AD. Introducing alternative energy sources, such as "biofuels," may also have the potential to alleviate bioenergetic dysfunction in AD [343]. Furthermore, a research investigation revealed that the application of asiaticoside extract significantly inhibited neuronal death in a rat model of rotenone-induced PD. The protective mechanism of asiaticoside on mitochondria involves safeguarding mitochondrial CI activity, which represents the rate-limiting step in OxPhos [350]. These findings indicate that asiaticoside holds promise for the treatment of neurodegenerative diseases, with a particular emphasis on AD, and has been extensively examined in clinical practice. Figure 4 illustrates the chemical structure of therapeutic candidates designed to target mitochondrial bioenergetics.

## Therapeutic Candidates Targeting Mitochondrial Biogenesis

**Figure 5.**
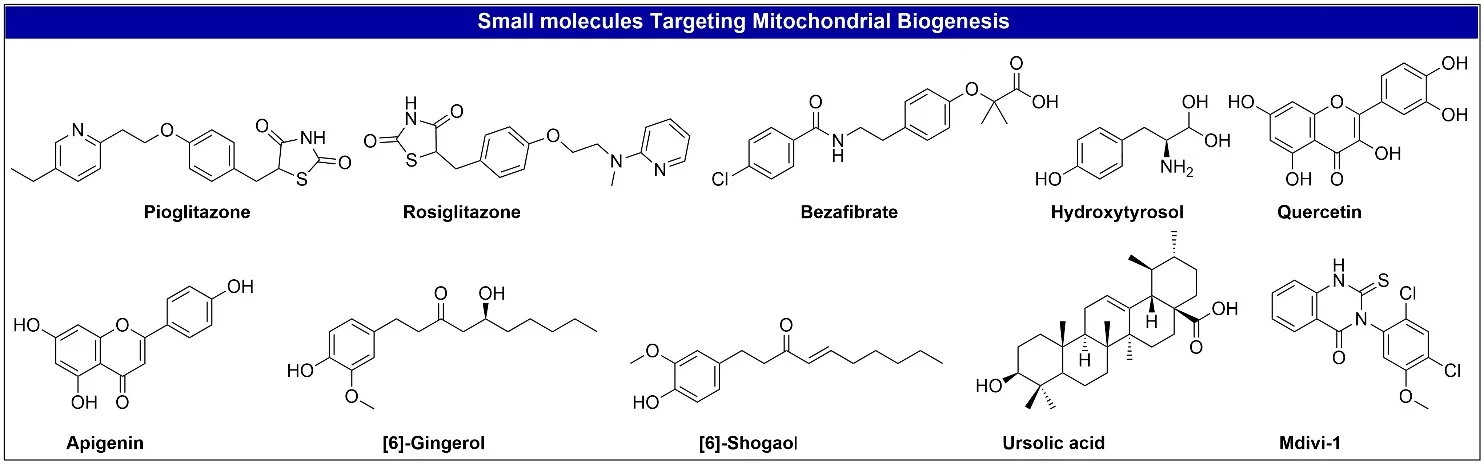
Chemical structure of therapeutic candidates targeting mitochondrial biogenesis.

Disturbed mitochondrial biogenesis and a decrease in the quantity of mtDNA are commonly observed in neurodegenerative disorders [351, 352]. The initiation of mitochondrial biogenesis plays a crucial role in maintaining the mitochondrial count, facilitating cell renewal, adapting to cellular damage, and meeting energy supply demands. Excessive expression of PGC-1α, the key regulator of mitochondrial biogenesis, can potentially mitigate mitochondrial damage and enhance biogenesis [330]. Additionally, PPARc can govern mitochondrial energy metabolism and stimulate mitochondrial biogenesis [353]. Consequently, strategic drug design focusing on PGC-1α and PPARc holds promise for the treatment of neurodegenerative disorders. Thiazolidinedione, assessed as a PPARc agonist, enhances cognitive function in AD patients by improving mitochondrial function [354]. Pioglitazone, an active compound categorized as a thiazolidinedione, decreases p-Tau and hippocampal Aβ levels and deactivates GSK3β in a cell line overexpressing the Tau protein [355]. Additionally, in a clinical trial investigating the impact of the PPARγ agonist pioglitazone on mild AD patients with type II diabetes mellitus, cognitive and functional enhancements were reported, as was disease stabilization in diabetic AD patients [356]. Notably, in this preliminary study, 3 patients in the pioglitazone group experienced mild peripheral edema as a negative side effect, but this adverse effect was tolerated without discontinuation of the drug candidate. Additional small molecules that activate the PPAR-PGC-1α axis include rosiglitazone and bezafibrate [357]. Rosiglitazone mitigates mitochondrial dysfunction by augmenting mitochondrial mass, whereas bezafibrate enhances mitochondrial ATP generation by enhancing mitochondrial protein expression [357]. A randomized, double-blind pilot trial with rosiglitazone revealed that a 6-month treatment with this PPAR agonist improved memory and selective attention [358]. This improvement was associated with a decrease in plasma Aβ40 and Aβ42 levels in twenty patients with AD compared to those in the control group [358]. Polyphenols, widely recognized for their antioxidant properties that counteract ROS, are pervasive in plant sources. In addition to their well-established antioxidant, anti-inflammatory, and cardiovascular preventive effects, certain polyphenols are now acknowledged for their ability to modulate molecules associated with mitochondrial biogenesis [359]. Given the direct or indirect involvement of mitochondrial dysfunction in the etiology of various neurodegenerative diseases, such as PD and AD, investigating the restoration of mitochondrial function is highly valuable [360]. Polyphenols, exemplified by resveratrol in grapes, hydroxytyrosol in olives, and quercetin in various plants, can induce mitochondrial biogenesis through the activation of the SIRT1/PGC-1 pathway [361-364]. Quercetin and resveratrol stimulate sirtuins and AMPK, thereby promoting mitochondrial biogenesis [357]. Resveratrol, in particular, has emerged as a promising candidate due to its well-known antioxidant, anti-inflammatory, and anti-apoptotic properties [365]. Resveratrol mitigates the levels of ROS and reactive nitrogen species by scavenging them, both of which impairs membrane lipids and DNA, mitochondrial functions and ultimately leading to neuronal damage and death [366, 367]. Resveratrol also stimulates the rise in endogenous anti-oxidant systems, such as HO-1, Nrf2, CAT, SOD, and GSH [368]. Furthermore, studies have suggested chronic resveratrol treatment significantly reduced the levels of oxidants and pro-inflammatory molecules [369, 370]. As a polyphenol found in red grapes, it stimulates AMPK and PGC-1α, activating SIRT1 and fostering mitochondrial biogenesis [371]. A phase II clinical study conducted in patients with mild to moderate AD demonstrated the safety and tolerability of resveratrol (NCT01504854). Thus, targeting dysregulated mitochondrial biogenesis through PGC-1α and PPARc represents a favorable approach for intervening in and treating AD. In APP/PS1 mice, a reduction in SIRT1 expression contributes to increased senile plaques and oxidative stress, a phenomenon partially reversed by the administration of resveratrol [333]. Apigenin, a primary flavonoid compound, and analogous substances promote adult neurogenesis by facilitating neuronal differentiation, suggesting that these compounds are beneficial for the treatment of neurodegenerative diseases. Interestingly, ginger extract and its primary constituents facilitate mitochondrial biogenesis in mouse cells, namely, [6]-gingerol and [6]-shogaol [372]. This effect is achieved by promoting OXPHOS subunit-related proteins and activating the AMPK-PGC1 signaling pathway [372]. Among various natural herbs, fruits, and vegetables, ursolic acid, a triterpenoid, stimulates mitochondrial biogenesis and ATP production [373, 374]. This occurs concurrently with minimal production of ROS in cells [373, 374]. ROS-induced stimulation of AMPK-PGC1 signaling within mitochondria improves the expression of cytochrome C oxidase (COX) and uncoupling protein 3, stimulating mitochondrial biogenesis [373, 374]. Figure 5 displays the chemical structure of therapeutic candidates aimed at enhancing mitochondrial biogenesis.

## Therapeutic Candidates Targeting Mitochondrial Dynamics

**Figure 6.**
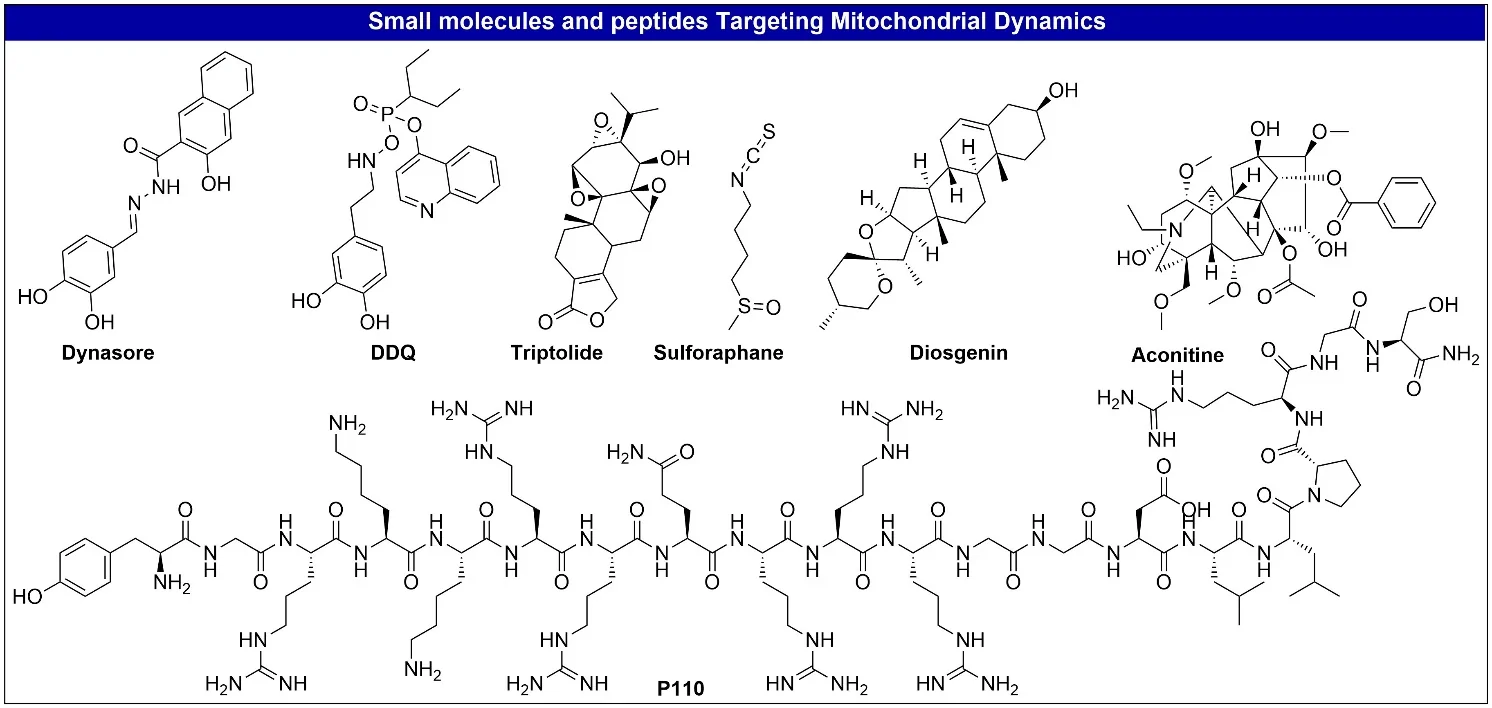
Chemical structure of therapeutic candidates targeting mitochondrial dynamics.

Various small molecules and peptides capable of inhibiting excessive mitochondrial fission have been identified and tested in cells and animals [375]. Mitochondrial division inhibitor 1 (mdivi-1) inhibits excessive mitochondrial fission and promotes fusion [376]. Mdivi-1 has therapeutic efficacy by alleviating Aβ toxicity and mitochondrial dysfunction in APP/PS1 double transgenic AD mice [377]. This treatment resulted in improved ATP production and enhanced neuronal viability. Notably, mdivi-1 administration prevents mitochondrial fragmentation in pyramidal neurons from CRND8 APP transgenic mice and hybrid AD cells, ensuring improved mitochondrial function [378]. Apart from directly addressing bioenergetic impairment, mdivi-1 has been shown to control neuroinflammation and moderately rescue mitochondrial damage associated with PINK1 inactivation [333]. Dynasore, recognized as a selective inhibitor that targets dynamin-1 and -2 and Drp1, has limited effectiveness on mitochondria, as reports indicate [379, 380]. The compound DDQ (diethyl (3,4-dihydroxyphenethylamino) (quinolin-4-yl) methylphosphonate) can reduce mitochondrial fission, enhance fusion, and induce mitochondrial biogenesis [381]. Sulforaphane, derived from cruciferous plants, modifies the dynamics of mitochondrial fusion and fission by inhibiting HDACs and DNA methyltransferases, thereby preventing genetic and epigenetic mutations [382]. Similarly, Diosgenin significantly diminished the loss of mitochondria-associated membrane structures that are crucial in the transfer of calcium from ER to associated mitochondrial network. Further, Diosgenin rescued mitochondrial functions (by the modulation of NDUFA4, a subunit of NADPH dehydrogenase, cytochrome c oxidase copper chaperon 17, and ATP synthase activity), following 3-MCPD toxicity [383]. Similarly, calenduloside E from Pterygoides auriculata, aconitine, the primary toxic compound of aconitine plants, targets OPA1 to induce the remodeling of mitochondrial function [384, 385]. Considering the pivotal role of mitochondria in neurons, these pharmaceutical agents may present potential therapeutic avenues for treating neurodegenerative diseases associated with disruptions in mitochondrial dynamics. The P110 peptide potently inhibits Drp1 activation by interrupting the Drp1/Fis1 interaction on the MOM [333]. Treatment with P110 in 5xFAD mice, which carry five AD-linked mutations, mitigates behavioral deficits, reduces Aβ buildup, addresses energy failure, and alleviates oxidative stress [53]. The integrity of mitochondrial structure and function was found to remain unaffected in cultured neuronal cells treated with amyloid beta and cells expressing APPswe (cells expressing Swedish amyloid precursor protein mutant form), as well as in five different fibroblasts derived from AD patients treated with P110 [53]. One of the main advantages of using P110 is that it is a selective Drp1 inhibitor and unlike other non-selective Drp-1 inhibitors (which broadly inhibit mitochondrial fission and have deleterious effects), it selectively blocks oxidative stress induced fission via Drp1-Fis1 interaction. This way, the drug preserves physiological homeostatic fission via Drp-1’s interaction with other adaptor proteins such as Mff, MiD49 and MiD51 [54]. Thus, P110 inhibits pathogenic mitochondrial fission in tissues without impacting basal fission (the physiological fission), even after prolonged in vivo use [377]. The concurrent application of P110 and mdivi-1 has demonstrated success in diverse preclinical models of neurodegenerative diseases, including AD, PD, and HD, and is undergoing further clinical investigation [375]. Figure 6 illustrates the chemical structure of therapeutic candidates designed to target mitochondrial dynamics.

## Therapeutic Candidates Targeting Mitophagy

**Figure 7.**
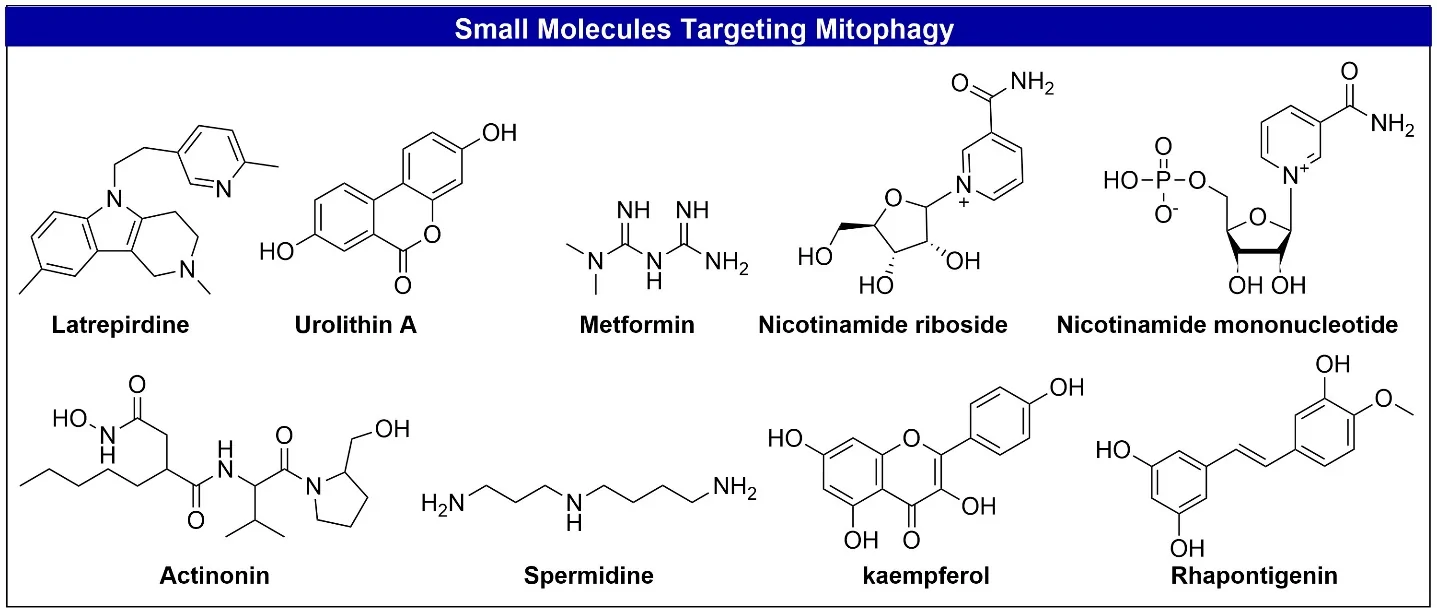
Chemical structure of therapeutic candidates targeting mitophagy.

Latrepirdine (Dimebon), an antihistamine drug, has demonstrated therapeutic efficacy against AD in various models by reducing mitochondrial swelling and stabilizing the ΔΨm [330]. The interplay between latrepirdine and glutamate receptors in a HEK cell overexpressing mutant APPs leads to blockade of calcium channels, inhibiting mitochondrial penetrability [386]. This inhibitory action suppresses unnecessary mitophagy or apoptosis, as latrepirdine markedly reduces the levels of mitochondrial permeability transition pore (mPTP) components, increasing the susceptibility of mitochondria to stressors. Consequently, targeting the inhibition of mPTP in mitophagy is regarded as a promising approach for developing new therapeutics. Latrepirdine demonstrated a significant improvement in cognitive function compared to a placebo in a phase II clinical trial involving patients with moderate AD [330]. However, a phase III clinical trial exploring the administration of Dimebon in mild to moderate AD patients was halted due to the absence of significant differences between the Dimebon and placebo groups (NCT00912288) [330]. In animal models, the natural food metabolite urolithin A (UA) has been demonstrated to restore neuronal mitophagy. UA eliminates AD-related Tau hyperphosphorylation, reduces ROS production, decreases insoluble Aβ42 and Aβ40, and mitigates cognitive impairment in APP/PS1 and 3xTg AD mice [387-389]. UA-induced mitophagy has been shown to significantly improve pathological aspects in vivo [390]. Studies have shown that treating *C. elegans* expressing Aβ1-42 and APP/PS1 mice with UA reduces Aβ pathology and cognitive decline through PINK1/Parkin-dependent mitophagy activation. UA preserves the structural and functional integrity of mitochondria and increases synaptic density [391]. Additionally, studies have shown that the mitophagy enhancer UA, alone or in combination with the green tea extract EGCG, is promising for treating late-onset AD [392]. The combination therapy improved mitochondrial dysfunction more strongly, reduced fragmented mitochondria, and increased mitophagosomal formation. The initial clinical trial conducted on sedentary elderly individuals confirmed the safety of UA at doses of 500 mg and 1000 mg over four weeks. This study also highlighted the beneficial effects of UA in modulating mitochondrial gene expression (NCT02655393) [393]. In addition to UA, both metformin and resveratrol, known regulators of PINK1/Parkin, also increase mitophagy [394, 395]. Metformin induces mitophagy by activating AMPK in vivo and in humans [390]. In the presence of Aβ, resveratrol enhances mitophagy in PC12 cells, demonstrating neuroprotective effects [396]. When administered orally for an extended period in APP/PS1 mice, resveratrol has been shown to improve memory and mitochondrial functions, activate SIRT1 and AMPK signaling, and reduce Aβ accumulation [390]. Resveratrol has significant clinical limitations due to its metabolic instability, which results in poor bioavailability [396]. While the role of SIRT10 in mitochondrial biogenesis has been established, a connection between SIRT1 and mitophagy was observed in 2009 when nicotinamide treatment accelerated the rate of mitophagic flux in primary human fibroblasts, extending their replicative lifespan [397]. This process enhances neuron survival and neurite growth [397]. Modulation of SIRT1 also promotes cognitive function and neurogenesis in the context of neurodegenerative disorders [330]. Thus, developing drugs that target or activate SIRT1 in dysfunctional mitochondria holds promise for intervening in and treating AD. NAD^+^, a cofactor for various proteins, including sirtuins such as SIRT1, 3, 6, and 7, plays a direct role in preserving the balance between mitochondrial biogenesis and mitophagy via NAD^+^-SIRT1-PGC1α signaling. A decrease in NAD^+^ levels reduces mitophagy and the accumulation of aggregated proteins in neurons [333]. NAD^+^ transporters, such as nicotinamide riboside and nicotinamide mononucleotide, are potent stimulators of mitophagy [390]. In *C. elegans* expressing Aβ1-42, nicotinamide riboside stimulates mitophagy, reducing Aβ accumulation and proteotoxic stress, ultimately extending survival [390]. In the same model, nicotinamide mononucleotide enhances PINK1/Parkin-dependent mitophagy and ameliorates memory defects [398]. In mice with APP/PS1, nicotinamide riboside has been shown to decrease cortical Aβ deposits; elevate the mRNA levels of PINK1, LC3, and OXPHOS proteins; and enhance cognitive functions [390]. Multiple clinical trials are currently underway to evaluate the impact of nicotinamide riboside on various aspects, such as brain function, cognition, oxidative stress, and CSF pTau levels, in individuals with mild cognitive impairment and AD (NCT02942888). Nevertheless, nicotinamide did not demonstrate a positive effect on cognitive function in a limited clinical study involving patients with mild to moderate AD [399]. Additionally, actinonin, an antibacterial compound, restores the morphology and functions of mitochondria and improves synaptic density in APP/PS1 mice by promoting mitophagy [390]. Spermidine, a natural product recognized for its ability to extend the lifetime of various organisms, including yeasts, flies, nematodes, and mice, achieves this by increasing autophagy [390]. Importantly, spermidine also activates PINK1/Parkin mitophagic signaling in human fibroblasts [390]. In a clinical trial involving elderly volunteers, spermidine was shown to enhance hippocampus-dependent memory (NCT02755246) [400]. Moreover, other natural products, kaempferol and rhapontigenin, are recognized for inducing mitophagy [390]. These molecules have been demonstrated to reduce p-Tau and Aβ levels and effectively ameliorate memory deficits both in vitro and in vivo [390]. Figure 7 shows the chemical structures of therapeutic candidates designed to target mitophagy. To summarize, numerous molecules can activate mitophagy, yielding beneficial effects in preclinical models of neurodegenerative diseases. However, there is a need to improve the activity of these therapeutics, with a specific focus on factors such as bioavailability, stability, ability to cross the BBB, pharmacokinetics, and interactions with their targets. This optimization process is crucial for advancing these potential treatments toward clinical application.

## Technological advancements in diagnosing and monitoring mitochondrial dysfunction in neurodegenerative diseases

Recent technological advancements in the diagnosis and monitoring of mitochondrial dysfunction in neurodegenerative diseases have been a focal point of research due to the critical role that mitochondrial health plays in these conditions. Neuroimaging methods such as positron emission tomography, magnetic resonance imaging, magnetic resonance spectroscopy, and near-infrared spectroscopy have been instrumental [401]. Currently a novel PET probe 2-*tert*-butyl-4-chloro-5-[6-(4-^18^F-fluorobutoxy)-pyridin-3-ylmethoxy]-2H-pyridazin -3-one (^18^F-BCPP-BF) has been used to visualize and assess the status of mitochondria in the brain [402]. This probe is intended to image mitochondria complex-I activity in living brain quantitatively by binding specifically with this complex with low nanomolar affinity [401-403]. Thus, in AD as well as in PD, impairments in mitochondria complex-I can be detected using this probe which corresponds to neuronal damage [398, 399]. Further, a highly potent technique for evaluating the metabolic signatures of mitochondrial dysfunction is mass spectrometry-based metabolomics [404]. Utilizing this technology, researchers have been able to examine the pathogenetic causes of mitochondrial metabolic disorders as well as the latest developments in their metabolomic remodelling [405]. For instance, compartment-specific metabolic signatures such as mutants of the succinate dehydrogenase (respiratory chain complex II) and of the F_O_F_1_-ATP-synthase (complex V) can be identified by applying metabolomics to isolated mitochondria and the corresponding cytoplasm [406]. These mutants of the mitochondrial oxidative phosphorylation machinery, in addition to altering citric acid cycle related metabolites, also have altered fatty acids, amino acids, purine and pyrimidine intermediates, ultimately leading to mitochondrial dysfunction [406]. Magnetic resonance imaging (MRI) studies are highly specific and sensitive in diagnosing mitochondrial diseases [407]. Using MRI, the chemical shift of metabolites as a marker of defective mitochondria has been quantified in neurodegeneration [408]. These metabolites include lactate, glycolysis end-product, creatine compounds, choline-containing compounds, myo-inositol, and N-acetyl-L-aspartate (NAA) [408].

The conventional techniques like qRT-PCR, immunoblotting, immunofluorescence, and seahorse bioanalysis are routinely being used to study and identify the mitochondrial anomalies and thus are a valuable tool for diagnosis and monitoring of neurodegenerative diseases like AD and PD [314, 409, 410]. For instance, a qRT-PCR study identified patients with PD had considerably altered expression levels of NDUFB7 mRNA and 3 DE-lncRNA, linked to mitochondrial respiratory activity [411]. Similarly another study identified METTL3 gene (encoding the subunit of N6-adenosine-methyltransferase) confers protection against mitochondrial dysfunction and cognitive impairment in APP/PS1 transgenic mice by upregulating Mfn2 via N6-methyladenosine modification [412]. Further a subsequent study identified MH84 (pirinixic acid derivative), mitigates mitochondrial dysfunction through PGC-1α dependent mechanism in Thy-1 AβPP_SL_ mice [413]. MH84 also mitigated the beta-secretase processing of amyloid precursor protein [413]. These findings highlight the potential of qRT-PCR as a tool for detecting and studying mitochondrial dysfunction in neurodegenerative diseases. Similarly, fluorescence-based methods for tracking and identifying a number of mitochondrial-based processes and functions—such as ATP content, iron metabolic accumulation, oxidative stress, mitochondrial fission and fusion, mitochondrial membrane potential, etc.—are rapid and affordable when investigating the role of mitochondria in neurodegenerative diseases [414-416]. One of the most widely used techniques to assess mitochondrial damage, for example, is mitotracker labeling in which a fluorescent dye attaches itself to the mitochondria of live cells to help assess the potential of the mitochondrial membrane [417, 418]. This method allows researchers to visualize and quantify changes in mitochondrial morphology and function, providing valuable insights into the progression of neurodegenerative diseases. Overall, the versatility and efficiency of these tools make them essential for advancing our knowledge of mitochondrial involvement in neurodegenerative disorders.

Seahorse flux analysis is a robust and economical approach for measuring cellular respiration and glycolysis in real-time [419]. By analyzing key parameters such as oxygen consumption rate and extracellular acidification rate, a deeper understanding of how mitochondria function in response to different stimuli during neurodegeneration can be obtained [419]. As such, seahorse flux analysis has become a crucial tool in studying the metabolic changes that occur in neurodegenerative diseases, offering a non-invasive and highly accurate method for monitoring mitochondrial activity [420]. By observing changes in mitochondrial function in real-time, we can track the progression of neurodegenerative diseases and potentially identify new targets for therapeutic intervention. This integrated approach provides a comprehensive understanding of how metabolic dysfunction contributes to neuronal damage in conditions such as AD and PD and may lead to the development of more effective treatments for these devastating disorders.

Traditional diagnosis methods such as PET and MRI are costly and also have a high chance of false-negative outcomes [421]. Moreover, they are time-consuming and not appropriate to detect mitochondrial DNA mutations [421]. To overcome these hurdles, sequencing methods are gaining popularity as the most reliable approach. Next-generation sequencing (NGS) is a rapid, cost-effective, and widely accessible identification method for the detection of mitochondrial mutational disorders [422-424]. About 1500 nuclear genes have been linked to mitochondrial function, and 15-20 new genes are discovered by NGS each year [425]. NGS allows for the simultaneous analysis of multiple genes, providing a comprehensive picture of a patient's genetic makeup in a timely manner [426]. This approach has significantly improved the accuracy and efficiency of diagnosing mitochondrial DNA mutations, leading to earlier interventions and improved outcomes for patients [426]. As our understanding of mitochondrial disorders continues to evolve, NGS will play a crucial role in advancing personalized medicine and enhancing the quality of life for individuals affected by these conditions. However, the main challenge in analyzing NGS data is accurately interpreting the large amount of genetic information generated, which requires advanced bioinformatics tools and expertise to distinguish between harmless variations and disease-causing mutations [427-430]. Nevertheless, the benefits of NGS in identifying and understanding mitochondrial DNA mutations outweigh the challenges, making it an invaluable tool in the field of genetic medicine. Furthermore, ongoing advancements in technology and data analysis methods are continually improving the accuracy and efficiency of NGS, further enhancing its potential to revolutionize the diagnosis and treatment of mitochondrial disorders [427-430].

## Conclusion and future perspectives:

The past decade has seen remarkable advancements in our understanding of mitochondrial dysfunction and its pivotal role in age-related neurodegenerative diseases. We conducted a comprehensive review examining the intricate relationship between mitochondrial dysfunction, excitotoxicity, oxytosis/ferroptosis, and neurodegeneration, with a particular focus on the substantial roles played by impaired mitochondrial biogenesis, oxidative stress, and compromised quality control mechanisms in age-related neurodegenerative diseases. This holistic understanding forms the foundation for potentially impactful therapeutic interventions, emphasizing the significance of targeting mitochondrial biogenesis and quality control mechanisms. The incorporation of small-molecule drugs and natural compounds as tools to modulate mitochondrial function and alleviate oxidative stress enriches our potential strategies for mitigating neurodegenerative processes.

To advance the development of more effective and targeted therapeutic interventions for neurodegenerative diseases, it is imperative to enhance our understanding of the molecular intricacies governing mitochondrial biogenesis and quality control processes. Identifying novel drug targets and refining delivery systems for small-molecule therapeutics and natural compounds will be crucial in achieving this goal. Furthermore, a deeper understanding of the complex interplay between excitotoxicity, oxytosis/ferroptosis, and mitochondrial dysfunction demands further exploration, potentially necessitating the creation of novel animal models and the utilization of advanced imaging techniques to study mitochondrial function and oxidative stress in vivo. This would not only drive our understanding of neurodegenerative diseases but also enhance the prospect of transformative therapeutic strategies for mitigating the complex interplay of mitochondrial dysfunction, excitotoxicity, and oxytosis/ferroptosis in age-related neurodegenerative pathologies.

Clinical studies targeting mitochondrial pathways in neurodegenerative diseases have not been very successful thus far. This limited success can be attributed to the overlooking of the complex interplay between various cellular systems, particularly the neuronal ER-mitochondria signaling, which is highly sensitive to homeostatic balance and whose disruption leads to imbalances in ATP production, axonal transport, lipid homeostasis, and synaptic dysfunction [431]. Additionally, challenges in drug delivery to the target site and the advanced disease stage in participants may hinder the restoration of mitochondrial function, thus impacting cellular health. The future of neuroprotective trials is likely to pivot towards personalized precision medicine, tailoring treatments based on individual genetic and environmental factors to address specific disruptions in cellular systems like ER-mitochondria signaling [431]. By targeting these specific pathways, we have a better chance at restoring homeostatic balance and ultimately improving outcomes for patients with neurodegenerative diseases. This individualized approach holds promise for more effective and successful treatments in the future. Moreover, employing strategies that target multiple pathways simultaneously may also prove beneficial in enhancing neuroprotection. Further, to accelerate the development of targeted therapies, interdisciplinary collaboration, data sharing, and the enrichment of study cohorts with diverse patient populations is necessary. By embracing this multifaceted approach, we can better address the heterogeneity of neurodegenerative diseases and improve outcomes for patients at every stage of their disease. Eventually, by combining cutting-edge research with a patient-centered focus, we can work towards comprehensive solutions that not only treat symptoms but also target the underlying causes of these devastating conditions. Additionally, ongoing research into novel biomarkers and imaging techniques may provide valuable insights into disease progression and treatment response, allowing for more accurate and timely interventions. As we continue to advance our understanding of the intricate mechanisms underlying neurodegeneration, the possibilities for innovative and personalized treatments will only continue to expand.

Among various molecules targeting mitochondria, Coenzyme Q10 and its derivatives remain extensively researched. However, clinical trials often fall short of achieving meaningful endpoints, partly due to the broad selection of enrolled neurodegenerative disease patients. To address pharmacodynamic and kinetic challenges, novel formulations and derivatives of CoQ10 have been developed [333]. Nevertheless, there are notable successes in clinical trials targeting mitochondrial pathways. Drugs like vatiquinone, now in a phase III trial, and CTI-1601, which has shown promising results in increasing FXN levels among patients with Friedreich ataxia, exemplify progress. Moreover, exploring potential candidates like resveratrol, recognized for its antioxidant properties, offers promise. However, the failure of some trials, such as Dimebon in treating mild to moderate AD, underscores the need for continued exploration and refinement of therapeutic approaches. Despite these challenges, there is optimism in overcoming obstacles by employing more precise biomarkers, selecting appropriate participants early in the disease progression, and implementing combined treatment strategies. Utilizing nanotechnology for targeted drug delivery to mitochondria represents a promising avenue for addressing neurodegenerative and metabolic diseases effectively.

## Supplementary Materials

The Supplementary data can be found online at: www.aginganddisease.org/EN/10.14336/AD.2024.0125-1.


