# Supplementary material for: Excitotoxicity, Oxytosis/Ferroptosis, and Neurodegeneration: Emerging Insights into Mitochondrial Mechanisms
Source: Aging Dis. 2024 Aug 1;16(5):2504–43. doi: 10.14336/AD.2024.0125-1 (PMC12339096; doi:10.14336/AD.2024.0125-1)
Supplement: Supplementary file 1 [file AD-16-5-2504-s.pdf]

SUPPLEMENTARY DATA

# Excitotoxicity, Oxytosis/Ferroptosis, and Neurodegeneration: Emerging Insights into Mitochondrial Mechanisms

**Sameera Khan, Nargis Bano, Shakir Ahamad, Urmilla John, Nawab John Dar, Shahnawaz Ali Bhat**

© 2024. Khan S et al. Published online at http://www.aginganddisease.org/EN/10.14336/AD.2024.0125-1

# SUPPLEMENTARY DATA

**Supplementary Table 1.** List of various clinical trials targetting mitochondrial dysfunction in neurodegenerative diseases along with their study status and phase involved with their primary/secondary outcomes

| ID | Study Title | Study status | Disease | Interventions and drugs | Phase | N | Primary/secondary outcome measures |
|---|---|---|---|---|---|---|---|
| **Clinical Trials against Alzheimer's Disease** | | | | | | | |
| NCT05617508 | A Dose Optimization Trial of Nicotinamide Riboside in Alzheimer's Disease | RECRUITING | Alzheimer's Disease | Nicotinamide Riboside | PHASE 2 | 80 | The between-visit difference in cerebral nicotinamide adenine dinucleotide (NAD) levels. Measured by 31P-Magnetic resonance spectroscopy (31P-MRS)/ The between-visit difference in cerebrospinal fluid (CSF) NAD and related metabolite levels/ The between-visit difference in cerebral metabolism patterns maximal alteration in the cerebral metabolism patterns/ The between-visit difference in the proportion of MRS responders |
| NCT04430517 | Effects of Nicotinamide Riboside on Bioenergetics and Oxidative Stress in Mild Cognitive Impairment/Alzheimer's Dementia | RECRUITING | Mild Cognitive Impairment/Mild Alzheimer Disease | Nicotinamide Riboside | EARLY PHASE 1 | 50 | Changes in brain NAD+ (redox levels)/ Changes in brain NAD+/NADH ratio/ Changes in brain CK/ATPase activity/ Changes in brain GSH levels |
| NCT05591027 | Safety and Target Engagement of Centella Asiatica in Cognitive Impairment | RECRUITING | Mild Cognitive Impairment/Alzheimer's Disease | Dried hot water extract (CAW) of Centella asiatica | PHASE 1 | 48 | N-acetylaspartate (NAA)/creatine (Cr) metabolite ratio (NAA/Cr)- indicator of neuronal viability and mitochondrial activity/ Ratio of 8-hydroxy-deoxyguanosine (8 OHdG) to creatinine in urine- measure of oxidative stress. |
| NCT04842552 | Effect of Hydralazine on Alzheimer's Disease | UNKNOWN | Alzheimer Disease | Hydralazine hydrochloride 25mg tablets | PHASE 3 | 424 | Various cognitive and function tests for patients and caregivers, olfactory tests, biochemistry as well as drug side effects will be assessed regularly over the period of follow-up. |
| NCT01354444 | Pilot Trial of Carvedilol in Alzheimer's Disease | Completed | Alzheimer Disease | Carvedilol | PHASE 4 | 29 | Hopkins Verbal Learning Test (HVLT) Scores at Baseline, 3, and 6 Months/ Effect of Carvedilol Treatment in Cerebrospinal Fluid (CSF) Levels of Amyloid-beta Oligomers/ |
| NCT02711683 | DL-3-n-butylphthalide Treatment in Patients with Mild to Moderate Alzheimer's Disease Already Receiving Donepezil: A Multi Centre, Prospective Cohort Study | Completed | Alzheimer Disease | DL-3-n-butylphthalide | N/A | 92 | Alzheimer's disease assessment scale-cognitive subscale (ADAS-cog)/ Clinician's Interview-Based Impression of Change Plus Caregiver Input (CIBIC-plus)/ Alzheimer's Disease Cooperative Study-Activities of Daily Living (ADCS-ADL)/ Neuropsychiatric Inventory (NPI) |
| NCT02017340 | A European Multicentre Double-blind Placebo-controlled Phase III Trial of Nilvadipine in Mild to Moderate Alzheimer's Disease | Completed | Alzheimer Disease | Nilvadipine | PHASE 3 | 511 | Alzheimer's Disease Assessment Scale (ADAS) Cog/ Clinical Dementia Rating Scale Sum of Boxes (CDR-sb)/ Disability Assessment for Dementia (DAD) |
| NCT03090516 | Clinical Study on Improving the Cognitive Function of Patients with Mild to Moderate Alzheimer's Disease by Using Ginkgo | Unknown | Alzheimer Disease | Ginkgo biloba dispersible tablets | PHASE 2 | 240 | Electroencephalography/ MMSE (Mini-mental State Examination)/ 1.5T MRI changes/ Alzheimer disease assessment scale (ADAS-cog)/ activities of daily living scale (ADL)/ Change in neuropsychiatric inventory (NPI) |

© 2024. Khan S et al. Published online at http://www.aginganddisease.org/EN/10.14336/AD.2024.0125-1

| | | | | | | | |
|---|---|---|---|---|---|---|---|
| | Biloba Dispersible Tablets | | | | | | |
| NCT02913664 | Exercise and Intensive Vascular Risk Reduction in Preventing Dementia | Completed | Alzheimer Disease | Angiotensin II receptor blocker (ARB, losartan) and calcium channel blocker (CCB, amlodipine) | PHASE 2 | 513 | Alzheimer's Disease Cooperative Study-Preclinical Alzheimer Cognitive Composite (ADCS-PACC) and NIH Toolbox (NIH-TB) Cognition Battery will be used to assess changes in neurocognitive function |
| NCT05383833 | Creatine to Augment Bioenergetics in Alzheimer's | RECRUITING | Alzheimer's Disease | Creatine Monohydrate: dietary supplement. | N/A | 20 | Adherence to Creatine Monohydrate Intervention/ Change in Blood Creatine/ Change in Brain Creatine Status/ Change in Cognition (NIH Toolbox (NIH-TB) Cognition Battery)/ Change in Peripheral Mitochondrial Respiration |
| NCT02460783 | Intermittent Calorie Restriction, Insulin Resistance, and Biomarkers of Brain Function | COMPLETED | Alzheimer's Disease/ Obesity/ Diabetes Mellitus | Boost (R) 5-2 diet/ Healthy Living Diet | N/A | 129 | Mean Change in Neuron-Derived Extracellular Vesicle (NDEV) Phosphorylated Serine312-insulin Receptor Substrate-1 (pS312-IRS-1)/ Mean Change in Neuron-Derived Extracellular Vesicle (NDEV) P-pan-Tyrosine-IRS-1 (pY-IRS-1)/ Mean Change in Body Mass Index (BMI) and weight. |
| NCT03101085 | S-Equol in Alzheimer's Disease 2 Trial (SEAD2) | COMPLETED | Alzheimer Disease | S-equol | PHASE 1 | 40 | Difference in cytochrome oxidase/citrate synthase (COX/CS) activity/ Montreal Cognitive Assessment, Alzheimer's Disease Assessment Scale-Cognitive Portion (ADASCog-11), Logical Memory Test (LMT) |
| NCT00678431 | A Single Centre, Multi-site, Randomized, Double-blind, Placebo-controlled Trial of Resveratrol with Glucose and Malate (RGM) to Slow the Progression of Alzheimer's Disease | COMPLETED | Alzheimer's Disease | Resveratrol with Glucose, and Malate: dietary supplement | PHASE 3 | 27 | Alzheimer Disease Assessment Scale (ADAScog)/ Clinical Global Impression of Change (**CGIC**) |
| NCT04044131 | A Phase 2, Randomized, Placebo Controlled Study to Evaluate the Efficacy, Tolerability and Safety of Metabolic Cofactor Supplementation in Alzheimer's Disease (AD) And | COMPLETED | Alzheimer Disease/ Parkinson Disease | For AD: Metabolic Cofactor Supplementation. For PD: Sorbitol | PHASE 2 | 120 | Mini Mental State Examination (MMSE)/ Alzheimer's Disease Assessment Scale-cognitive subscale (ADAS-cog)/ Alzheimer's Disease Cooperative Study - Activities of Daily Living (ADCS-ADL)/ Unified Parkinson's Disease Rating Scale (UPDRS) |

| | | | | | | | |
|---|---|---|---|---|---|---|---|
| | Parkinson's Disease (PD) Patients | | | | | | |
| NCT05040321 | A Proof of Concept Trial of a Sirtuin-NAD Activator in Alzheimer's Disease | RECRUITING | Alzheimer's Disease /Dementia | MIB-626 | PHASE1/PHASE2 | 50 | change in CSF concentrations of MIB-626/ change in CSF concentrations of MIB-626 metabolites, nicotinamide (NAM), NR, 2-PY, and MeNAM/ change in the abundance of NAD in the brain using ultra-high field 7T magnetic resonance spectroscopy |
| NCT03514875 | Effects of Mitochondrial-targeted Antioxidant on Carotid Artery Endothelial Function and Brain Blood Flow in Mild Cognitive Impairment (MCI) Patients | WITHDRAWN (discontinued due to change in operating plans prior to study initiation and enrollment) | Alzheimer Disease, Early Onset/Mild Cognitive Impairment | MitoQ: dietary supplement | N/A | 0 | Carotid artery blood flow/ Oxidative Stress/ Cerebrovascular Oxygenation/ Brain Electrical Activity/ Endothelial Function |
| NCT03702816 | The Relationship Between Neuropsychological Testing and MRI, PET and Blood Biomarkers in Neurodegenerative Disease (COBRE - Project 1): AIM 2 | TERMINATED (GE180 has limited Blood-brain barrier permeation reducing its utility in permeation. Also, its start date was under COVID-19 Thus creating supply chain issues, impacting enrollment). | Alzheimer Disease/ Parkinson Disease/ Inflammation | GE180 PET Scan | PHASE 2 | 24 | Frontal, Cingulate, Parietal, Temporal, Whole Brain GE180 Standardized Uptake Value Ratio (SUVR)/ Memory, Executive Function, Speed, Language Composite Score (Z-score)/ Dementia Rating Score/ Montreal Cognitive Assessment Score (MoCA) |
| NCT04018092 | Revitalizing Cognition in Older Adults at Risk for Alzheimer's Disease with Near-Infrared Photobiomodulation | RECRUITING | Cognitive Aging/Alzheimer Disease | Device: Active NIR-PBM | PHASE 2 | 168 | Change in Active group ARENA (spatial navigation task, a human analogue to the Morris WaterMaze) scores compared to Sham group ARENA scores |
| NCT03860792 | Therapeutic Diets in Alzheimer's Disease | RECRUITING | Alzheimer Disease | Ketogenic Diet/ Therapeutic Lifestyles Changes Diet | N/A | 80 | Change in cognitive performance on the Alzheimer's Disease Assessment Scale Cognitive Subscale (ADASCog11), Mini-Mental State Exam (MMSE), Logical Memory Test (LMT) and by Stroop test/ Change in Clinical Dementia Rating (CDR) |
| NCT00951834 | Sunphenon EGCG (Epigallocatechin-Gallate) in the Early Stage of Alzheimer´s Disease | COMPLETED | Alzheimer's Disease | Epigallocatechin-Gallate | PHASE2/PHASE3 | 21 | ADAS-COG (Score 0-70)/ MMSE (Score 0-30) after 18 months compared to baseline/ Safety and tolerability of the verum/ Brain atrophy assessed by brain MRI |
| NCT04098666 | Metformin in Alzheimer's Dementia Prevention | Recruiting | Alzheimer's Disease | Extended release metformin: oral | PHASE2/PHASE3 | 326 | Free and Cued Selective Reminding Test (FCSRT)/ Alzheimer's Disease Cooperative Study Preclinical Alzheimer's Cognitive Composite (PACC-ADCS)/ Cortical Thickness/ White matter hyper intensity volume (WMH)/ Brain amyloid; tau/ Complex I activity |
| NCT00829374 | CONCERT: A PHASE 3 Multicentre, Randomized, Placebo-Controlled, Double-Blind Twelve-Month Safety and Efficacy Study Evaluating Dimebon in Patients | COMPLETED | Alzheimer's Disease | Dimebon | PHASE 3 | 1003 | Alzheimer's Disease Cooperative Study - Activities of Daily Living (ADCS-ADL)/ Alzheimer's Disease Assessment Scale - Cognitive Subscale/ Clinician's Interview Based Impression of Change, plus caregiver input (CIBIC-plus)/ Neuropsychiatric Inventory (NPI)/ Resource Utilization in Dementia Lite (RUD lite)/ Euro Quality of Life 5 (EQ-5D) |

© 2024. Khan S et al. Published online at http://www.aginganddisease.org/EN/10.14336/AD.2024.0125-1

| | | | | | | | |
|---|---|---|---|---|---|---|---|
| | with Mild-to-Moderate Alzheimer's Disease on Donepezil | | | | | | |
| NCT00099710 | A PHASE II, Double-Blind, Placebo-Controlled Study of the Safety and Tolerability of Two Doses of Curcumin C3 Complex Versus Placebo in Patients with Mild to Moderate Alzheimer's Disease | Completed | Alzheimer's Disease | Curcumin C3 Complex: dietary supplement | PHASE 2 | 33 | Oxidative damage/ I nflammation/gliosis/ A-beta levels/ Tau levels/ Total plasma cholesterol (LDL,HDL,ApoE)/ Plasma curcumin and metabolites/ Cognitive and behavioral measures |
| NCT00675623 | A Global Phase 3, Double-Blind, Placebo-Controlled Safety and Efficacy Study of Oral Dimebon in Patients with Mild-to-Moderate Alzheimer's Disease (CONNECTION) | COMPLETED | Alzheimer's Disease | Dimebon | PHASE 3 | 598 | To determine the effect of Dimebon as compared to placebo on the primary measure of cognition and memory (ADAS-cog), (CIBIC-plus), (ADCS-ADL) and behaviour by Neuropsychiatric Inventory (NPI) |
| NCT01388478 | Safety/Tolerability and Effects on Cognitive Impairment, Impaired Cerebral Cortical Metabolism and Oxidative Stress of R(+)Pramipexole Administered to Subjects With Early Alzheimer's Disease | COMPLETED | Alzheimer's Disease | R-pramipexole | PHASE 2 | 20 | Number of Patients with Adverse Events/ Effects on Cognitive Performance/ Changes in Cerebral Glucose Metabolism/ Reduction of Oxidative Stress |
| NCT02142777 | S-Equol in Alzheimer's Disease (SEAD) Trial | COMPLETED | Alzheimer's Disease | S -Equol | PHASE 1 | 15 | Platelet mitochondria cytochrome oxidase (COX) activity/ safety of S-equol [Time Frame: 6 weeks] |
| NCT04740580 | Glutathione, Brain Metabolism and Inflammation in Alzheimer's Disease | RECRUITING | Alzheimer's Disease | Glycine, N-acetylcysteine, Alanine | EARLY PHASE1 | | Cognition (Measured using ADAS-Cog testing)/ Brain glucose uptake/ Brain inflammation/ Mitochondrial fuel oxidation/ Damage due to oxidative stress/ Inflammatory cytokines/ Plasma concentration of Brain-derived neurotropic factor (BDNF)/ Mitochondrial energetics. |
| NCT05929924 | Does EVOO Induce Gene and Metabolic Changes in Healthy Subjects with Alzheimer's Disease Family History | NOT_YET_RECRUITING | Alzheimer Disease | Extra virgin olive oil: dietary supplement | N/A | 40 | Changes in the concentrations of blood metabolites (metabolomics)/ Changes in the concentrations of blood mRNA transcripts (transcriptomics) |
| NCT04701957 | The Ketogenic Diet for Alzheimer's Disease: A Randomized Controlled Feasibility Study. | RECRUITING | Alzheimer Disease, Early Onset | Ketogenic diet | N/A | 70 | Feasibility of Ketogenic diet with urinary ketone levels / Safety of ketogenic diet with weight, albumin levels and lipid levels/ Efficiency of ketogenic diet on cognition (MMSE test) |
| NCT02062099 | PET Imaging of the Translocator Protein Ligands (TSPO) With [18 F] DPA-714 Biomarker of NeuroInflammation in Cognitive Decline (NIDECO) | COMPLETED | Memory Complaint/Mild Cognitive Impairment/Alzheimer Disease | [18F]DPA-714 PET/ [18F]AV-45 PET/neuropsychological assessment | PHASE 1 | 25 | Fixation and distribution of [18F] DPA-714 (Binding Potential BP)/ [18F] AV-45 Standard Uptake Values ratio/ Relationship between [18F] DPA-714 uptake and cognitive, affective symptoms at baseline |

© 2024. Khan S et al. Published online at http://www.aginganddisease.org/EN/10.14336/AD.2024.0125-1

| | | | | | | | |
|---|---|---|---|---|---|---|---|
| NCT05343611 | Combining Vitamin E-functionalized chocolate With Physical Exercise to Reduce the risk Of Protein Energy Malnutrition in Pre-dementia Aged People | RECRUITING | Dementia/Dementia, Mild/Dementia Moderate/Dementia Senile/Malnutrition/Deficiency Nutritional/Deficiency Diseases | Combination of High Protein Diet and Physical Exercise protocol/ Participants add to their diet 30 grams of 85% dark chocolate high in polyphenols, functionalized with 100 mg of Vitamin E per day | N/A | 102 | Change in free-fat soft tissue mass (g) |
| NCT05081219 | Study of Nasal Insulin to Fight Forgetfulness - Combination Intranasal Insulin and Empagliflozin Trial | RECRUITING | Mild Cognitive Impairment/Cognitive Impairment/Alzheimer Disease | Insulin/ Empagliflozin 10 mg. Device: Aptar Pharma CPS Intranasal Delivery Device | PHASE 2 | 60 | Number of Participants with Treatment-related Serious Adverse Events as Assessed by CTCAE v5.0/ Change in the Preclinical Alzheimer Cognitive Composite 5 (PACC5) Z-Score/ Change in the 14-item Alzheimer's Disease Assessment Scale-Cognitive subscale (ADAS-Cog 14) Score/ Change in amyloid β-peptide (Aβ40,42) in Cerebrospinal Fluid (CSF) along with levels of total tau and phospho-tau 181 |
| **Clinical Trials against Parkinson's Disease** | | | | | | | |
| NCT05344404 | NR-SAFE: A Safety Study Investigating Treatment with High-dose Nicotinamide Riboside (NR) in Parkinson's Disease | COMPLETED | Parkinson Disease | Nicotinamide Riboside: dietary supplement | N/A | 20 | Incidence of treatment-associated moderate and severe adverse events (AEs)/ Between-group (NR vs placebo) difference in changes of the NAD metabolome in blood and urine, measured by mass spectrometry (LC-MS/MS Q-Exactive HF) |
| NCT03568968 | A Randomized Controlled Trial of Nicotinamide Riboside Supplementation in Early Parkinson's Disease: the NOPARK Study | RECRUITING | Parkinson Disease | Nicotinamide Riboside: dietary supplement | PHASE 3 | 400 | Disease severity assessed by the total MDS-UPDRS (Movement Disorder Society Unified Parkinson's Disease rating Scale) subsections I-III/ Change in the severity of nigrostriatal degeneration assessed by [$^{123}$I] FP-CIT single photon emission CT, non-motor symptoms assessed by the Non-Motor Symptoms Assessment Scale, cognitive decline assessed by the Montreal Cognitive Assessment (MoCA) scale |
| NCT03840005 | A Phase II, Placebo Controlled, Double Blind, Randomised Clinical Trial to Assess the Safety and Tolerability Of 30mg/kg Daily Ursodeoxycholic Acid (UDCA) In Patients with Parkinson's Disease (PD) | COMPLETED | Parkinson's Disease | Ursonorm | PHASE 2 | 31 | Number of Participants with Incidence of Treatment-Emergent Adverse Events/Participants with Incidence of Serious Adverse Events/Participants that complete the study |
| NCT04287543 | Effect of Melatonin Administration on the PER1 and BMAL1 Clock Genes in Patients | WITHDRAWN (due to COVID-19 pandemic they were unable to start the study) | Parkinson Disease | Melatonin | PHASE2/PHASE3 | 0 | Expression levels of clock genes/ SCOPA-Sleep scale/ Epworth scale/ Anxiety/ Depression/ Activity of the mitochondrial complex 1/ Oxidative stress |

© 2024. Khan S et al. Published online at http://www.aginganddisease.org/EN/10.14336/AD.2024.0125-1

| | | | | | | | |
|---|---|---|---|---|---|---|---|
| | with Parkinson's Disease | | | | | | |
| NCT00329056 | A Double-Blind, Prospective, Randomized Comparison of 2 Doses of MitoQ and Placebo for the Treatment of Patients with Parkinson's Disease | COMPLETED | Parkinson's Disease | MitoQ | PHASE 2 | 128 | Unified Parkinson's Disease Rating Scale (UPDRS) score at the final study visit compared to baseline/ UPDRS sub scores/ MMSE/ Schwab and England Scale/ Modified Hoehn and Yahr Scale/ Timed tapping score |
| NCT05589766 | N-DOSE: A Dose Optimization Trial of Nicotinamide Riboside in Parkinson's Disease | RECRUITING | Parkinson Disease | Nicotinamide Riboside: dietary supplement | PHASE 2 | 80 | The between-visit difference in cerebral NAD levels/ The between-visit difference in CSF NAD and related metabolite levels/ between-visit difference in expression of the Nicotinamide Riboside Related Pattern (NRRP)/ The between-visit difference in the proportion of MRS responders |
| NCT00517842 | A Triple-blinded, Randomised, Placebo-controlled Trial to Examine the Efficacy and Safety of ViNeuro in Patients with Parkinson's Disease | COMPLETED | Parkinson Disease | ViNeuro | N/A | 160 | The primary efficacy outcome is the change from baseline in the sum of the Unified Parkinson's Disease Rating Scale (UPDRS) (Appendix 6) Parts II and III total scores at the end of 24 weeks. The UPDRS is to be performed one hour after L-dopa treatment |
| NCT01364545 | Ketogenic Diets for Symptoms of Parkinson's Disease | UNKNOWN | Parkinson's Disease | Ketone ester drink: dietary supplement | N/A | 20 | Unified Parkinson's Disease rating Scale, part III (motor)/ Timed motor tasks as per CAPSIT/ Computerised reaction time and cognitive tests. |
| NCT03457493 | UAB Neuroinflammation in Parkinson's Disease - TSPO-PET Substudy | RECRUITING | Parkinson Disease | DPA-714-PET/MRI | PHASE1/PHASE2 | 205 | Comparison of TSPO-PET measures of neuroinflammation between PD patients and healthy controls/ Correlation of DPA-714-PET/MRI with demographics, clinical and biospecimen assessments from Neuroinflammation in PD study |
| NCT02967250 | 7T Magnetic Resonance Spectroscopy Monitoring Brain Bioenergetics in Parkinson's Disease and Response to Repeated Oral UDCA Treatment | COMPLETED | Parkinson Disease | ursodeoxycholic acid | PHASE 1 | 5 | Change in ATP concentration using 7T MRS/ UDCA pharmacokinetics |
| NCT05855577 | Clinical Efficacy of Pharmacological Treatments Targeting Energy Metabolism, Evaluated by Gait Analysis, on Motor Function in Parkinson's Disease Patients | NOT_YET_RECRUITING | Parkinson Disease/ Gait Analysis /Therapy, Directly Observed/Metabolic Disease | Terazosin | PHASE 4 | 50 | Clinical evaluation, Gait Analysis and Metabolic variables efficacy of therapy/ The efficacy and molecular mechanisms of Nrf2 pathway modulation in PD rodent models |
| NCT02462603 | A Phase 2A Safety and Biomarker Study of EPI-589 in Mitochondrial Subtype and Idiopathic Parkinson's Disease Subjects | COMPLETED | Parkinson's Disease | PTC-589 | PHASE 2 | 44 | Number of Participants With Drug-Related Serious Adverse Events (SAEs)/ Change From Baseline in Movement Disorder Society Sponsored Revision of the Unified Parkinson's Disease Rating Scale (MDS-UPDRS) Score at Month 3/ Change From Baseline in Non-motor Symptoms Scale (NMSS) Total Score at Month 3/ Change From Baseline in |

| | | | | | | | |
|---|---|---|---|---|---|---|---|
| | | | | | | | Parkinson's Disease Questionnaire - 39 (PDQ-39) Score at Month 3/ Change From Baseline in EuroQol-5 Dimension (EQ-5D) Score at Month 3/ Montreal Cognitive Assessment (MoCA) Score/ Beck Depression Inventory (BDI) Score |
| NCT05963425 | The Effects on Physical Activity on Mitochondrial Function in Skin Fibroblasts in Patients with Parkinson's Disease: A Study Protocol | RECRUITING | Parkinson's Disease | Physical activity | N/A | 24 | Change in oxygen consumption rate/ Change in ATPmax levels/ Non-motor and motor function/ Sleepiness/ Mobility/ Cognitive aspects/ Mood |
| NCT05214287 | An N-of-1 Double-blind Randomized Phase 1 Trial of the Safety and Feasibility of (Intermittent) Hypoxia Therapy in Parkinson's Disease (TALISMAN) | COMPLETED | Parkinson’s Disease | Hypoxic Gas Mixture | PHASE1/PHASE2 | 29 | Nature and number of adverse events/ Self-reported dizziness, discomfort and stress on a ten-point scale/ Blood pressure/ Heartrate/ Respiratory rate/ Oxygen saturation/ Feasibility questionnaire |
| NCT04768023 | Influence of 12 Weeks Vitamin D Supplementation Combined with Physical Activity on Blood and Functional Parameters and Quality of Life in Parkinson's Disease Patients Treated with Deep Brain Stimulation | COMPLETED | Vitamin D Deficiency/Parkinson Disease | Juvit D3 | N/A | 50 | The effects of vitamin D supplementation and physical activity on concentration of vitamin D3 in serum, concentration of inflammatory markers in serum, concentration of CRP in serum, concentration of kynurenine pathway metabolites in serum, - the evaluation of changes before and after 12 weeks of supplementation and physical activity. 6-minute walk test and Up & Go and 10-meter walk test |
| NCT04477161 | Effect of Ketone Esters on Parkinson Disease: A Pilot, Prospective Trial | COMPLETED | Parkinson Disease/ Ketosis | Ketone Ester Elite Endurance Nutrition Drink: dietary supplement | N/A | 10 | Changes in serum Ketones |
| NCT03061513 | Ubiquinol in Parkinson's Disease: Safety, Tolerability, and Effects Upon Oxidative Damage and Mitochondrial Biomarkers | COMPLETED | Parkinson Disease | Ubiquinol: dietary supplement | PHASE 2 | 11 | Number of Adverse Events/ Cerebral Redox Markers |
| **Clinical Trials against Huntington’s Disease** | | | | | | | |
| NCT00712426 | Creatine Safety, Tolerability, & Efficacy in Huntington's Disease (CREST-E) | TERMINATED (results of interim analysis showed that creatine was ineffective in slowing down the loss of function in early symptomatic HD) | Huntington's Disease | Creatine Monohydrate | PHASE 3 | 553 | Change in Total Functional Capacity/ Clinical symptoms (changes in other UHDRS scores); safety (frequency of adverse events); tolerability (proportion of subjects completing study at assigned dosage level), quality of life, other biological markers |
| NCT01879267 | Exercise Effects in Huntington's Disease | COMPLETED | Huntington's Disease | BEHAVIORAL: Exercise training | N/A | 40 | Change in Unified Huntington's Disease Rating Scale (UHDRS) |

© 2024. Khan S et al. Published online at http://www.aginganddisease.org/EN/10.14336/AD.2024.0125-1

# SUPPLEMENTARY DATA

| | | | | | | | |
|---|---|---|---|---|---|---|---|
| NCT01882062 | Proof of Concept of an Anaplerotic Study Using Brain Phosphorus Magnetic Resonance Spectroscopy in Huntington Disease | COMPLETED | Huntington Disease | Triheptanoin 1g/kg/day | PHASE 2 | 10 | Ratio of Inorganic Phosphate (Pi) Over Phosphocreatine (PCr): Pi/PCr/ |
| NCT01502046 | A Double Blind, Randomized, Cross Over, Placebo Controlled Phase 2 Clinical Trial to Asses Neuroprotection by Cannabinoids in Huntington's Disease | COMPLETED | Huntington's Disease | delta-9-tetrahydrocannabinol (THC) and cannabidiol (CBD) | PHASE 2 | 25 | Serious Adverse Events reported/ Changes in the UHDRs Score/ Changes in the BDNF levels (Brain-derived Neurotrophic Factor), oxidative stress (due to mitochondrial dysfunction) and proinflammatory cytokines in CSF and plasma |
| **Clinical Trials against Amyotrophic Lateral Sclerosis** | | | | | | | |
| NCT02874209 | Non-invasive Assessment of Neuronal Damage by MRI Sodium (23Na) in Amyotrophic Lateral Sclerosis | UNKNOWN | Amyotrophic Lateral Sclerosis | sodium MRI | N/A | 60 | Central conduction time of the potential muscle through transcranial magnetic stimulation |
| NCT00005766 | Clinical Trial of Creatine in Amyotrophic Lateral Sclerosis | COMPLETED | Amyotrophic Lateral Sclerosis | Creatinine | PHASE 2 | | |
| NCT04244630 | Mitochondrial Capacity Boost in ALS (MICABO-ALS) Trial | RECRUITING | Amyotrophic Lateral Sclerosis | COMBINATION_PRODUCT: Antioxidants | PHASE 2 | 60 | Measurement of serum Neurofilament light chain (NfL)/ Measurement of functional decline in ALS/ Frequency of serious adverse events and adverse events/ Survival analysis |
| NCT02969759 | Bioenergetics and Protein Metabolism in Sporadic Amyotrophic Lateral Sclerosis | UNKNOWN | Sporadic Amyotrophic Lateral Sclerosis | Skin Biopsy | EARLY PHASE 1 | 30 | Kinetics of fibroblast growth/ Mitochondrial metabolism/ Protéic metabolism/ stress in senescence |
| NCT01232738 | A Multi-Center Controlled Screening Trial of Safety and Efficacy of Rasagiline in Subjects with Amyotrophic Lateral Sclerosis (ALS) | COMPLETED | Amyotrophic Lateral Sclerosis | rasagiline | PHASE 2 | 36 | Amyotrophic Lateral Sclerosis Functional Rating Scale - Revised (ALSFRS-R)/ Difference in Time to Treatment Failure |
| NCT03506425 | A Pilot Trial of Triheptanoin for People with Amyotrophic Lateral Sclerosis (PALS) | COMPLETED | Amyotrophic Lateral Sclerosis | Triheptanoin | PHASE1/PHASE2 | 15 | ALS Functional Rating Scale-revised Version (ALSFRS-R) Slope/ Change in NAA/Cr Ratio from Motor Cortex as Measured by Magnetic Resonance Spectroscopy/ Change in Urine Isoprostane Levels, an Oxidative Stress Marker |
| NCT01854294 | GM604 Phase 2A Randomized Double-blind Placebo Controlled Pilot Trial in Amyotrophic Lateral Disease (ALS) | COMPLETED | Amyotrophic Lateral Sclerosis | GM604 | PHASE 2 | 12 | Efficacy by percent change in biomarker in the CSF at week 12 from baseline/ Safety by measuring 1. adverse event frequency and severity, changes in vital signs, clinical laboratory values. 2. Serious adverse event frequency/ Tolerability by measuring the ability to complete the first 2 weeks of active treatment in the study |
| NCT04140136 | The Efficacy and Safety of Vitamin E Mixed Tocotrienols In Patients with | UNKNOWN | Amyotrophic Lateral Sclerosis | Tocotrienols: dietary supplement | PHASE 2 | 20 | Mean change of revised ALS Functional Rating Scale (ALSFRS-R) at baseline and 6 months between treatment group difference/ Number of participants with |

© 2024. Khan S et al. Published online at http://www.aginganddisease.org/EN/10.14336/AD.2024.0125-1

# SUPPLEMENTARY DATA

| | | | | | | | |
|---|---|---|---|---|---|---|---|
| | Amyotrophic Lateral Sclerosis (ALS): A Pilot Exploratory Study | | | | | | treatment-related adverse events, haematological, renal and liver profile monitored at every visit |
| NCT00243932 | Clinical Trial of High Dose CoQ10 in ALS | COMPLETED | Amyotrophic Lateral Sclerosis \|Lou Gehrig's Disease | coenzyme Q10 | PHASE 2 | 185 | Change in the ALS Functional Rating Scale-revised (ALSFRSr) Score/ The Change Over 9 Months in Forced Vital Capacity; Fatigue Severity Scale; Short Form-36; and 8OH2dG |
| NCT04820478 | Efficacy and Tolerability of Beta Hydroxybutyrate Ester in Patients with Amyotrophic Lateral Sclerosis (ALS) | RECRUITING | Amyotrophic Lateral Sclerosis | Beta Hydroxybutyrate Ester (KetoneAid KE4): dietary supplement | N/A | 76 | Neurofilament Light Chain (NfL) serum levels/ Survival/ Amyotrophic Lateral Sclerosis Functional Rating Scale Revised/ Body Mass Index/ Slow Vital Capacity/ Resting Energy Expenditure |

N/A: Data not available

© 2024. Khan S et al. Published online at http://www.aginganddisease.org/EN/10.14336/AD.2024.0125-1
